# The wucai genome and DNA methylation regulation on the inner leaves’ yellowing response to low temperature

**DOI:** 10.1093/hr/uhaf231

**Published:** 2025-09-02

**Authors:** Lingyun Yuan, Jian Wang, Haoying Zhang, Jinfeng Hou, Wenjie Wang, Xun Gao, Yating Yang, Gaoxia Wang, Min Li, Jianqiang Wu, Shidong Zhu, Guohu Chen, Xiaoyan Tang, Xinyu Xie, Chenggang Wang

**Affiliations:** Vegetable Genetics and Breeding Laboratory, College of Horticulture, Anhui Agricultural University, Hefei 230036, China; Anhui Provincial Engineering Laboratory of Horticultural Crop Breeding, Hefei 230036, China; Department of Vegetable Culture and Breeding, Wanjiang Vegetable Industrial Technology Institute, Maanshan 238200, China; Vegetable Genetics and Breeding Laboratory, College of Horticulture, Anhui Agricultural University, Hefei 230036, China; Anhui Provincial Engineering Laboratory of Horticultural Crop Breeding, Hefei 230036, China; Vegetable Genetics and Breeding Laboratory, College of Horticulture, Anhui Agricultural University, Hefei 230036, China; Anhui Provincial Engineering Laboratory of Horticultural Crop Breeding, Hefei 230036, China; Vegetable Genetics and Breeding Laboratory, College of Horticulture, Anhui Agricultural University, Hefei 230036, China; Anhui Provincial Engineering Laboratory of Horticultural Crop Breeding, Hefei 230036, China; Department of Vegetable Culture and Breeding, Wanjiang Vegetable Industrial Technology Institute, Maanshan 238200, China; Vegetable Genetics and Breeding Laboratory, College of Horticulture, Anhui Agricultural University, Hefei 230036, China; Anhui Provincial Engineering Laboratory of Horticultural Crop Breeding, Hefei 230036, China; Department of Vegetable Culture and Breeding, Wanjiang Vegetable Industrial Technology Institute, Maanshan 238200, China; Vegetable Genetics and Breeding Laboratory, College of Horticulture, Anhui Agricultural University, Hefei 230036, China; Anhui Provincial Engineering Laboratory of Horticultural Crop Breeding, Hefei 230036, China; Vegetable Genetics and Breeding Laboratory, College of Horticulture, Anhui Agricultural University, Hefei 230036, China; Anhui Provincial Engineering Laboratory of Horticultural Crop Breeding, Hefei 230036, China; Vegetable Genetics and Breeding Laboratory, College of Horticulture, Anhui Agricultural University, Hefei 230036, China; Anhui Provincial Engineering Laboratory of Horticultural Crop Breeding, Hefei 230036, China; Vegetable Genetics and Breeding Laboratory, College of Horticulture, Anhui Agricultural University, Hefei 230036, China; Anhui Provincial Engineering Laboratory of Horticultural Crop Breeding, Hefei 230036, China; Vegetable Genetics and Breeding Laboratory, College of Horticulture, Anhui Agricultural University, Hefei 230036, China; Anhui Provincial Engineering Laboratory of Horticultural Crop Breeding, Hefei 230036, China; Department of Vegetable Culture and Breeding, Wanjiang Vegetable Industrial Technology Institute, Maanshan 238200, China; Vegetable Genetics and Breeding Laboratory, College of Horticulture, Anhui Agricultural University, Hefei 230036, China; Anhui Provincial Engineering Laboratory of Horticultural Crop Breeding, Hefei 230036, China; Department of Vegetable Culture and Breeding, Wanjiang Vegetable Industrial Technology Institute, Maanshan 238200, China; Vegetable Genetics and Breeding Laboratory, College of Horticulture, Anhui Agricultural University, Hefei 230036, China; Anhui Provincial Engineering Laboratory of Horticultural Crop Breeding, Hefei 230036, China; Department of Vegetable Culture and Breeding, Wanjiang Vegetable Industrial Technology Institute, Maanshan 238200, China; Vegetable Genetics and Breeding Laboratory, College of Horticulture, Anhui Agricultural University, Hefei 230036, China; Anhui Provincial Engineering Laboratory of Horticultural Crop Breeding, Hefei 230036, China; Department of Vegetable Culture and Breeding, Wanjiang Vegetable Industrial Technology Institute, Maanshan 238200, China; Vegetable Genetics and Breeding Laboratory, College of Horticulture, Anhui Agricultural University, Hefei 230036, China; Anhui Provincial Engineering Laboratory of Horticultural Crop Breeding, Hefei 230036, China; Vegetable Genetics and Breeding Laboratory, College of Horticulture, Anhui Agricultural University, Hefei 230036, China; Anhui Provincial Engineering Laboratory of Horticultural Crop Breeding, Hefei 230036, China; Department of Vegetable Culture and Breeding, Wanjiang Vegetable Industrial Technology Institute, Maanshan 238200, China

## Abstract

Wucai (*Brassica rapa*), a widely cultivated non-heading Chinese cabbage in autumn and winter in China, has been limited in its genetic and genomic analyses due to the lack of a reference genome. Here, we have assembled a high-quality chromosome-level genome assembly of wucai, which is 480.57 Mb in length, with a scaffold N50 of 46.53 Mb and a contig N50 of 4.45 Mb. We annotated 42 634 protein-coding genes in the *B*. *rapa* W7-2 genome and 55.08% of repetitive sequences. Additionally, we performed DNA methylome analysis to investigate the characteristic yellowing response of wucai inner leaves to low temperature. CHH methylation levels increased under low-temperature conditions, while a slight decrease was observed under normal temperatures with more hypo-differentially methylated regions. The expression levels of DNA methyltransferases *BrCMT2* and *BrDRM2* were significantly up-regulated under low-temperature conditions but down-regulated at normal temperature. Furthermore, chlorophyll metabolism genes, *BrHemA*, *BrHemL*, *BrHemD*, *BrCLH2*, *BrCHLP*, *BrSGR*, and *BrPPD*, were identified as differentially expressed, which exhibited elevated CHH methylation levels in their promoter regions across three stages. Leaves of *Nicotiana benthamiana* transiently overexpressing *BrCLH2.1* exhibited a slight degreening phenotype compared to wild type. The application of a DNA methylation inhibitor into the inner leaves of W7-2 induced obvious bleaching, and the total chlorophyll content decreased significantly. In conclusion, the genome data of W7-2 provide a valuable resource for functional gene research and genetic breeding of non-heading Chinese cabbage.

## Introduction

The *Brassica* crop genus features a classic evolutionary model known as the ‘triangle of U’, which involves three diploid members: *Brassica rapa* (AA, 2*n* = 20), *Brassica nigra* (BB, 2*n* = 16), and *Brassica oleracea* (CC, 2*n* = 18), whose pairwise genomic combinations generate three distinct allopolyploids. The resultant tetraploid species include *Brassica napus* (AACC, 2*n* = 38) from AA × CC crosses, *Brassica juncea* (AABB, 2*n* = 36) originating from AA × BB hybridization, and *Brassica carinata* (BBCC, 2*n* = 34) formed through BB × CC chromosomal fusion [1]. The Brassicaceae lineage exhibits a unique whole-genome triplication (WGT) event that has shaped genomic evolution across *Brassica* species [2]. Subsequent convergent selection acting on paralogous genes in subgenomes has driven substantial phenotypic diversification [3]. Comparative analyses of multiple high-quality genomes have revealed structural variations—including presence–absence variants (PAVs), structural variants (SVs), and copy number variants (CNVs)—that drive these genomic diversification processes [4–6]. Elucidating genomic variation patterns reveals the molecular basis of agriculturally important traits, facilitating marker discovery for precision breeding strategies in *Brassica* crop improvement.

Many *Brassica* species and subspecies, including Chinese cabbage, turnip, and pak choi, display considerable phenotypic variation [7, 8]. Prolonged selection during domestication has driven striking morphological divergence in *Brassica* crops, manifesting as specialized structures, including leafy head formation, hypertrophy of storage organs (roots/stems/inflorescences), and architecturally complex axillary branching patterns [9]. Traditionally, research on *B. rapa* crops has predominantly centered on Chinese cabbage and pak choi, while wucai is considered a variant of non-heading cabbage. Previous studies have successfully constructed the *B. rapa* pan-genome, delineating core and dispensable genes while also identifying SVs [10]. To date, the three published draft genomes of *B*. *rapa* ssp. *chinensis* cultivars, *B*. *rapa* PC-fu [11], *B*. *rapa* NHCC001 [12] and pak choi with purple leaves [13], which belong to the non-heading Chinese cabbage group, have enabled rapid progress in the discovery of new genes and the characterization of genomic variation, including the identification of heat-resistant genes BrcCER1, heading genes, PAV, and SV. These findings provide fundamental insights into evolutionary trajectories of genome architecture and polygenic trait regulation mechanisms.

Wucai, also a form of non-heading Chinese cabbage, has been one of the most popular leafy vegetables in the Yangtze-Huai River basin of China for more than 1000 years. It is adapted to cultivation in open fields in autumn and winter. Notably, its typical genotype displays temperature-dependent color transition; the inner leaves shift from green to yellow as temperatures gradually decline in autumn and winter [14, 15]. This phenotype is a unique trait of wucai responding to low temperature, which distinguishes it from the other varieties of non-heading Chinese cabbage. Previous studies on leaf color in non-heading Chinese cabbage have identified a stable genetic yellow-leaf mutant, *pylm*, derived from the ‘Huaguan’ cultivar [16]. Additionally, pak choi with purple leaves has been reported [17], and a novel orange-headed Chinese cabbage mutant regulated by a single recessive gene, *Br-or*, has been characterized, which exhibits the ability to accumulate carotenoids [18]. To date, no research has been reported on the low-temperature-induced yellowing of inner leaves in non-heading Chinese cabbage. Therefore, wucai is a potentially valuable germplasm resource for analyzing the genetic basis of traits and economically important traits. Chromosome-level assemblies of wucai enable systematic deciphering of molecular determinants underlying its agronomically valuable characteristics, including leaf yellowing, cold tolerance, and leaf curling.

Here, using Hi-C technology, we generated a chromosome-scale diploid (2*n* = 2*x* = 20) genome assembly for *B. rapa* ssp. *chinensis* cultivar wucai. This high-quality reference genome enabled a better understanding of the genetic basis of wucai evolution. By combining transcriptome and DNA methylome analysis, we investigated the dynamic patterns of DNA methylation during temperature-induced inner-leaf yellowing in wucai and analyzed the relationship between differentially expressed genes involved in porphyrin and chlorophyll metabolic pathways and their association with DNA methylation changes. These findings underscore the significance of a high-quality reference genome in deciphering complex genomic architectures, while providing essential resources to advance molecular breeding in wucai.

## Results

### The *B*. *rapa* W7-2 genome assembly, validation, and annotation

A typical wucai cultivar ‘W7-2’ was subjected to sequencing. The genome of *B*. *rapa* W7-2 was evaluated using Illumina short reads and single-molecule nanopore long-read sequencing, yielding ~64.01 Gb (131.9 × genome coverage), and 62.96 Gb of clean reads was obtained for survey analysis (Table S1). *K*-mer analysis showed that the *B*. *rapa* W7-2 genome was approximately 474.91 Mb, with 0.393% heterozygosity and 52.83% repetitive sequences (Fig. S1 and Table S2).

To obtain a high-quality genome assembly, we generated 508.2 Gb of raw sequencing data, and 1 146 595 valid reads totaling 17.46 Gb (37×) were obtained after processing (Table S3). The W7-2 genome assembly was improved using Hi-C sequencing, yielding a final assembly size of 480.57 Mb with a 46.53 Mb scaffold N50 and 4.45 Mb contig N50 (Table S4). In total, 437.62 Mb of sequences were anchored to 10 superscaffolds with an anchor rate of 91.06% (Table S5). The Hi-C interaction heatmap reveals that the final chromosome-scale genome of wucai contains 10 clusters (Fig. S2). To evaluate the accuracy and genomic integrity, benchmark universal single copy homologous sequences (BUSCO) analysis showed an assembly quality of 99.3% (Tables S6 and S7).

Protein-coding genes in *B*. *rapa* W7-2 were annotated through an integrated approach combining transcriptome, homology-based, and *de novo* prediction methods. A total of 42 634 protein-coding genes were annotated in *B*. *rapa* W7-2, of which 42 548 (99.80%) were homologous to at least one entry in a public protein database (Fig. S3 and Table S8).

Functional annotation through Gene Ontology (GO) and Kyoto Encyclopedia of Genes and Genomes (KEGG) enrichment analyses elucidated conserved metabolic pathways in the *B*. *rapa* W7-2 genome (Fig. S3B and D). And 2.74% (13.16 Mb) of the assembled genome was annotated as noncoding RNA (Table S9). A total of 469 227 isoforms were detected with a mean length of 1075 bp (Table S10). BLASR was used to identify 27 894 reference genes (refgenes) (67.65% of the genome), with 12.67 mean isoforms for each gene (Table S11). Meanwhile, a total of 207 fusion genes, 10 314 known transcripts, 26 693 known alternative splicing (AS) events, and 25 385 new transcripts were detected across all samples in *B*. *rapa* W7-2 (Table S12).

**Figure 1:**
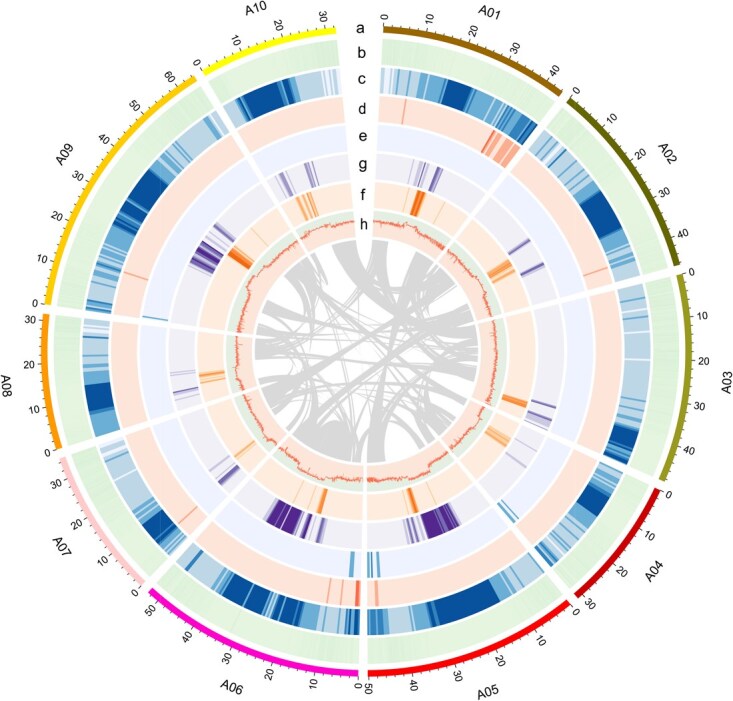
Characteristics of the *B*. *rapa* W7-2 genome. (a) Chromosome length; (b) per-chromosome gene density; (c) per-chromosome TE content; (d) DNA density; (e) LINE density; (f) Gypsy retrotransposons density; (g) Copia retrotransposons density; (h) per-chromosome distribution of GC content. The inner lines indicate syntenic blocks in the genome.

A total of 264.76 Mb (55.08%) of the *B*. *rapa* W7-2 assembly sequences were annotated as repetitive sequences (Table S13) with 51.49% transposable elements (TEs), including DNA, long interspersed nuclear elements (LINEs), short interspersed nuclear elements (SINEs), long terminal repeats (LTRs), and other types. Similar to the pattern observed in most plant genomes [19], LTRs emerged as the dominant TE class, spanning 134.44 Mb and accounting for 27.97% of the assembled genome (Table S14). DNA transposons and LINEs accounted for only 7.36% and 3.74% of the W7-2 genome, respectively (Table S14). These TEs display stochastic chromosomal distribution patterns inversely correlated with gene density (Fig. 1). The length and numbers of LTRs in *B*. *rapa* W7-2 were close to those in *B*. *rapa* PC-fu (126.3 Mb, 77.6%) and almost triple those in *B*. *rapa* Chiifu (57.8 Mb, 54.6%) [11, 20].

The BUSCO analysis completely covered 1496 conserved proteins in the gene set, with a completeness of 92.69% and a missing fraction of 2.11%, of which 1161 (71.93%) were present as single copies and 335 (20.76%) as multiple copies (Table S15). This result indicated that the gene annotation integrity of *B*. *rapa* W7-2 was close to that of other published non-heading Chinese cabbage varieties.

### *Brassica rapa* W7-2 genome evolution and chromosome homology

**Figure 2:**
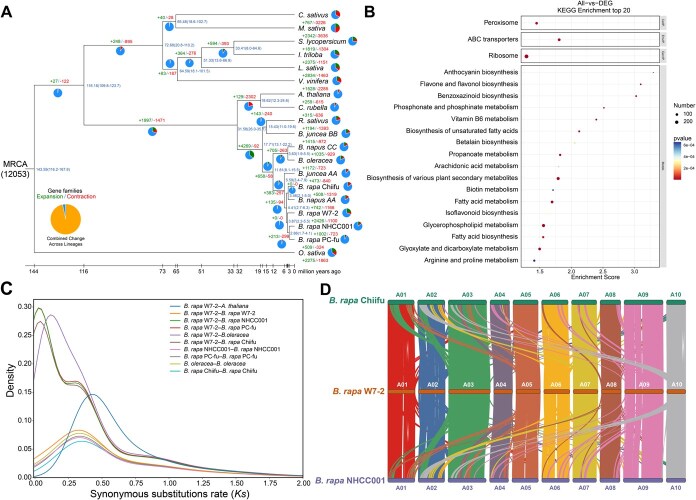
Comparative analysis of the *B*. *rapa* W7-2 Genome. (A) Gene family dynamics across 19 species. Color-coded numerals denote evolutionary changes: green (expanded) and red (contracted). (B) Significant KEGG pathways enriched in expanded genes of B. rapa W7-2. (C) Synonymous substitution rates (*Ks*) distribution across collinear gene pairs in *B*. *rapa* W7-2 compared with related species. (D) Genomic collinearity between three *Brassica* crops.

To explore the genome evolutionary history of *B. rapa* W7-2, gene family clustering was carried out using *B. rapa* W7-2 and 18 other species (Table S16). We identified 43 425 gene families in total according to the clustering results (Table S17). Species divergence time estimation (Fig. 2A) and phylogenetic reconstruction (Fig. S4) were performed using single-copy orthologs shared by 19 plants identified through gene family clustering. The *Brassica* ancestor diverged from *A*. *thaliana* ~31.58 million years ago (Mya), with subsequent intraspecies differentiation events in *B*. *rapa* W7-2 diverged from Chinese cabbage (*B*. *rapa* Chiifu) and non-heading Chinese cabbage (*B*. *rapa* NHCC001, *B*. *rapa* PC-fu) estimated to be approximately 4.41 and 3.87 Mya, respectively (Fig. 2A). In *B. rapa* W7-2, there were 2426 and 1100 expanded and contracted orthogroups, respectively (Fig. 2A). Interestingly, 2426 expanded orthogroups (10 575 genes) were enriched in the biosynthesis of complex secondary metabolites, including some pathways related to stress tolerance, such as biosynthesis of unsaturated fatty acids, fatty acid biosynthesis, phosphonate and phosphinate metabolism, and vitamin B6 metabolism (Fig. 2B). Therefore, the functions of these expanded genes in the *B. rapa* W7-2 genome suggested that the genes facilitate high stress resistance in wucai.

Among the 19 genomes, the numbers of single-copy genes and multiple-copy genes in the four *B. rapa* crops were similar, but *B*. *rapa* PC-fu harbored more unique genes than the other three *B. rapa* plants (Figs S5 and S6). A total of 15 940 common orthogroups were found among the four species. The number (685) of *B. rapa* W7-2-specific orthogroups exceeded that of *B. rapa* NHCC001 (460) but was less than those of the other two species (Fig. S5). These 685 specific orthogroups for *B. rapa* W7-2 included 916 genes. Functional analysis of these 916 genes showed enrichment in numerous metabolic pathways, such as brassinosteroid biosynthesis, alpha−linolenic acid metabolism, galactose metabolism, fatty acid degradation, and carbon fixation in photosynthetic organisms (Fig. S7). According to our previous study, the taxon-specific genes and expanded orthogroups enriched in these pathways may play an important role in regulating the cold tolerance of wucai [21–23].

The synonymous substitution rates (*Ks*) among the five *Brassica* crops indicated that a recent WGT event occurred at *Ks* = 0.32 in the evolutionary history of *B. rapa* W7-2 (Fig. 2C). The divergence of *B. rapa* W7-2, *B*. *rapa* Chiifu, *B*. *rapa* PC-fu, and *B*. *rapa* NHCC001 occurred at *Ks* = 0.02, followed by that of *B. rapa* W7-2 and *B. oleracea* (*Ks* = 0.1) (Fig. 2C). The 10 chromosomes of *B. rapa* W7-2 showed strong homology with those of the other two species (*B*. *rapa* Chiifu, *B*. *rapa* NHCC001), and the collinearity relationship has been maintained at a high level, indicating close evolutionary relationships among the three plants (Fig. 2D). Further analysis of structural variation showed that there were 13.29% (63.87 Mb) sequence differences between *B*. *rapa* Chiifu and *B*. *rapa* W7-2, encompassing single nucleotide polymorphisms, insertion/deletion variants (InDels), CNVs, highly divergent regions (HDRs), and tandem repeats (TRs) (Table S17). Duplications (DUPs) represented the most prevalent SV type, affecting 14.33% (68.86 Mb) of *B*. *rapa* W7-2 genome (Fig. S8 and Table S18). A sequence difference of 10.94% (52.58 Mb) was detected between *B*. *rapa* W7-2 and *B*. *rapa* NHCC001 (Table S19). Translocations (TRA) constituted the dominant structural variation type, impacting 8.56% (41.15 Mb) of *B*. *rapa* W7-2 genome (Fig. S8 and Table S20).

### DNA methylation dynamics of the wucai whole-genome response to low temperature

**Figure 3:**
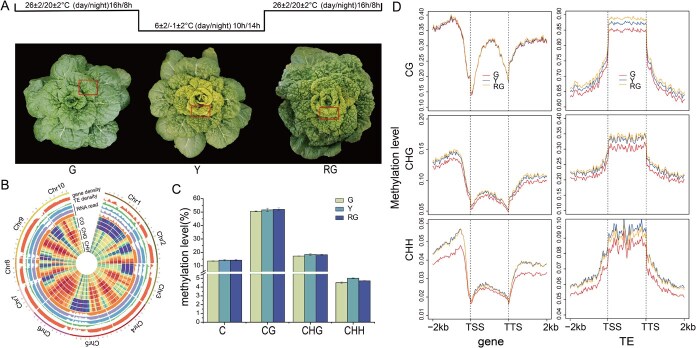
DNA methylation landscape of the wucai genome during inner-leaf yellowing. (A) Single plant phenotype of wucai in G, Y, and RG. Inner leaves: inside the box, this location is the sampling site. (B) Chromosomal architecture visualization of *B*. *rapa* W7-2 genome using Circos plots. Track order: gene density, TE density, RNA read in G, Y, and RG. CG, CHG, and CHH methylation levels in G, Y, and RG. (C) Average methylation levels of mC, mCG, mCHG, and mCHH in wucai leaves in G, Y, and RG. (D) DNA methylation profiles of CG, CHG, and CHH related to genes (left) and TEs (right).

As a typical trait of wucai, the inner leaves displayed a yellowish color during autumn and winter under low temperature (Fig. 3A). To explore the DNA methylation landscape response to temperature, we performed whole-genome methylation maps of wucai under normal and low temperature. The DNA methylomes for low temperature before color change (G), low temperature after color change (Y), and normal temperature after color change (RG) obtained by whole genome bisulfite sequencing (WGBS) had an average 22× sequencing depth and 20.871 Gb of clean reads per sample (Table S21). Approximately 50% of the reads were uniquely mapped to the wucai reference genome using BSMAP, and the conversion rates were >99.24% (Table S22). At the genome scale, there was a low methylation level and expression level in the TE-rich regions in G, Y, and RG stages. In contrast, the gene-rich regions showed a high methylation level and expression level in G, Y, and RG stages (Fig. 3B). The wucai genome showed percentages of 46% (mCG), 26% (mCHG), and 28% (mCHH) in the G stage. Compared to the G stage, the proportion of mCG and mCHG decreased by 1%, while mCHH increased by 2% in the Y stage. In the RG stage, the trend was similar to that in the G stage (Fig. S11).

In this study, comparative methylation analysis across three developmental stages (G, Y, RG) revealed distinct patterns: CG contexts exhibited the highest methylation levels, whereas CHH contexts showed the lowest. Compared to the G stage, the methylation levels of the three contexts were increased in the Y. However, the methylation level of the CHH decreased in the RG stage, while that of CG and CHG contexts did not change (Fig. 3B). The whole-genome methylation levels of three stages were profiled across six elements in genomic regions: Up2k, CDS, Down2k, mRNA, CpG island, and repeat. The repeat region, CpG island region, and Up2k region have relatively high methylation levels, suggesting these loci function as epigenetic regulatory elements potentially modulating transcriptional activity (Fig. S12).

Inner-leaf yellowing in wucai correlated with pronounced DNA methylation plasticity across temperature regimes. To further investigate DNA methylation dynamics of the wucai whole-genome response to low temperature, we analyzed the average DNA methylation levels for genes and TEs. High methylation levels were found in the flanking regions of genes and the body regions of TEs. During the Y developmental stage, CG, CHG, and CHH contexts collectively exhibited elevated methylation levels. However, in the RG stage, the CHH methylation level was slightly decreased in the promoter and body regions of genes and flanking and body regions of TEs (Fig. 3D).

### Methylation characterization of genes undergoing methylation response to different temperatures

**Figure 4:**
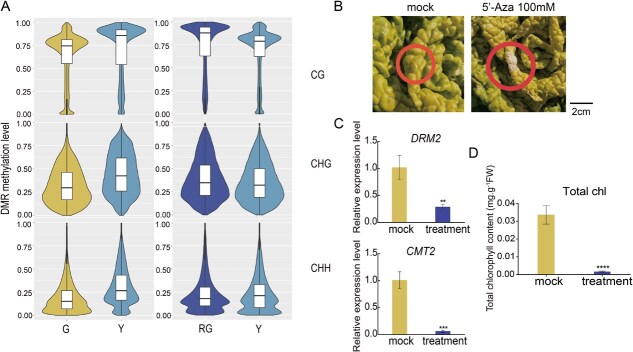
Methylation characterization of genes undergoing methylation response to different temperatures. (A) Violin boxplots showing DNA methylation levels of DMRs in G, Y, and RG. Methylation levels in CG, CHG, and CHH contexts are shown. TSS, transcription start site; TTS, transcription termination site. (B) Photographs of wucai treated with DNA methylation inhibitor, 100-Aza, or mock (ddH_2_O). White bar, 2 cm. The treated areas are circled. (C) The expression of *DRM2* and *CMT2* in mock and 100-Aza-treated wucai inner leaf. (D) Chlorophyll content in mock and 100-Aza-treated wucai inner leaf. Data were derived from six biological replicates of each period. Error bars represent ± SD. Different numbers of * above the bars indicate significant differences (^*^P < 0.05, ^**^P < 0.01, ^***^P < 0.001, ^****^P < 0.0001).

Differentially methylated genes were classified into two subgroups: differentially methylated regions (DMR)-associated genes and DMR-associated promoters. There were 23 118 DMR genes and 32 627 DMR promoters in Y/G, and there were 17 193 DMR genes and 25 622 DMR promoters in RG/Y. Furthermore, the number of hyper-DMRs was higher than the number of hypo-DMRs (Table S23). In DMR-associated genes, most hyper-DMRs and hypo-DMRs occurred in the CG context. In contrast, most hyper-DMRs and hypo-DMRs occurred in the CHH context in DMR-associated promoters. In the comparison of Y/G and RG/Y subgroups, the number of hyper-DMRs gradually decreased in the CG context, but the number of hypo-DMRs in RG/Y increased significantly only in the CHG and CHH contexts, particularly in CHH content (Fig. S13). This may be the main reason for the decrease in CHH methylation levels in the RG stage. A comparative analysis revealed that DMRs of the three contexts in the Y stage showed higher methylation levels than those in the G stage, but only the CHH context in the RG stage displayed lower methylation levels than that in the Y stage (Fig. 4A). Most hyper-DMRs and hypo-DMRs were distributed in the CHH promoter regions in RG/Y (Fig. S14). The above results showed that CHH methylation in promoter regions plays a key role in wucai inner-leaf yellowing.

To obtain more information about how DNA methylation affects inner-leaf yellowing, the transcript levels of epigenetic modifier genes (DNA methyltransferases/demethylases) were profiled. In the wucai genome, we identified four DNA methyltransferases, BrCMT2 (CHROMOMETHYLASE 2), BrMET (METHYLTRANSFERASE), BrDRM1 (DOMAINS REARRANGED METHYLTRANSFERASE 1), and BrDRM2 (DOMAINS REARRANGED METHYLTRANSFERASE 2) (Fig. S15A). Among them, *BrCMT2* and *BrDRM2* mainly contribute to CHH methylation. Of these four genes, *BrDRM1* showed no difference in expression in the three stages, and *BrMET1.2* showed no significant difference in the Y stage. Compared with those in the G stage, the expression levels of *BrCMT2* and *BrDRM2* in the Y stage significantly increased, and those in the RG stage declined (Fig. S15B). Additionally, the *BrDML1* (*Demeter-like protein 1*) expression increased progressively from the G to RG stages. The expression of *BrDML3* (*Demeter-like protein 3*) increased significantly in the Y stage but was not changed in the RG stage (Fig. S15D). According to the above results, the expression changes of *BrCMT2* and *BrDRM2* were consistent with the change in DNA methylation levels associated with inner-leaf yellowing. Therefore, we speculated that *BrCMT2* and *BrDRM2* might cause changes in DNA methylation levels.

To further analyze the significance of DNA methylation for inner-leaf yellowing, DNA methylation inhibitor 5-azacytidine was injected into the inner leaves of W7-2. The result showed that the application of a DNA methylation inhibitor into the inner leaves of W7-2 induced obvious bleaching, the expression levels of *BrDRM2* and *BrCMT2* had decreased, and the total chlorophyll content decreased significantly (Fig. 4B–D). These results support the role of DNA hypomethylation in wucai inner-leaf yellowing.

### Correlation analysis between DNA methylation levels and gene expression levels during inner-leaf yellowing in wucai

To explore the dynamic coupling between transcriptional regulation and epigenetic modifications during inner-leaf yellowing, the transcriptome libraries of G, Y, and RG stages were constructed and sequenced. After filtering, 42.62M, 42.67M, and 43.37M clean reads were identified in G, Y, and RG libraries, respectively. The filtered base numbers were 6.39, 6.4, and 6.50 Gb, respectively (Table S24). Transcriptome comparisons were performed for Y/G and RG/Y to identify differentially expressed genes (*P* < 0.05 and |log2FC| > 1). DEGs (2344 up-regulated and 2977 down-regulated) were identified for Y/G, and DEGs (4195 up-regulated and 3729 down-regulated) were identified for RG/Y (Fig. S16). Through comparing the DEGs of the two groups of Y/G and RG/Y, 2802 DEGs were co-expressed in the two groups (Fig. S17). Hierarchical clustering of 2802 DEGs revealed bidirectional regulation signatures between Y/G and RG/Y, and the expression level of these genes showed opposite trends in the two groups (Fig. S18).

Previous studies indicated that genomic methylation changes may be caused by CHH methylation in promoter regions. To investigate the relationship between CHH methylation changes in the promoter regions and gene expression, we identified DMR-associated promoters among the overlapping DMRs and DEGs. We found that the CHH-DMR-associated DEGs accounted for at least 60% of the total number of DMR-associated DEGs (Fig. S19A). To explore how CHH methylation changes affect gene expression, we analyzed DMRs (hyper-DMR and hypo-DMR) and DEGs (up-regulated and down-regulated). By comparing the DEGs identified in the Y/G and RG/Y comparisons, we identified 711 hyper-DMR-down-regulated DEGs, 561 hyper-DMR-up-regulated DEGs, 75 hypo-DMR-up-regulated DEGs, and 125 hypo-DMR-down-regulated DEGs in Y/G and 411 hyper-DMR-down-regulated DEGs, 475 hyper-DMR-up-regulated DEGs, 410 hypo-DMR-up-regulated DEGs, and 391 hypo-DMR-down-regulated DEGs in RG/Y (Fig. S19B and Table S25). KEGG enrichment analysis showed that only DEG-Up-DMR-hypo in Y/G and DEG-Down-DMR-hyper in RG/Y were enriched in porphyrin and chlorophyll metabolic pathways (Fig. S19C).

**Figure 5:**
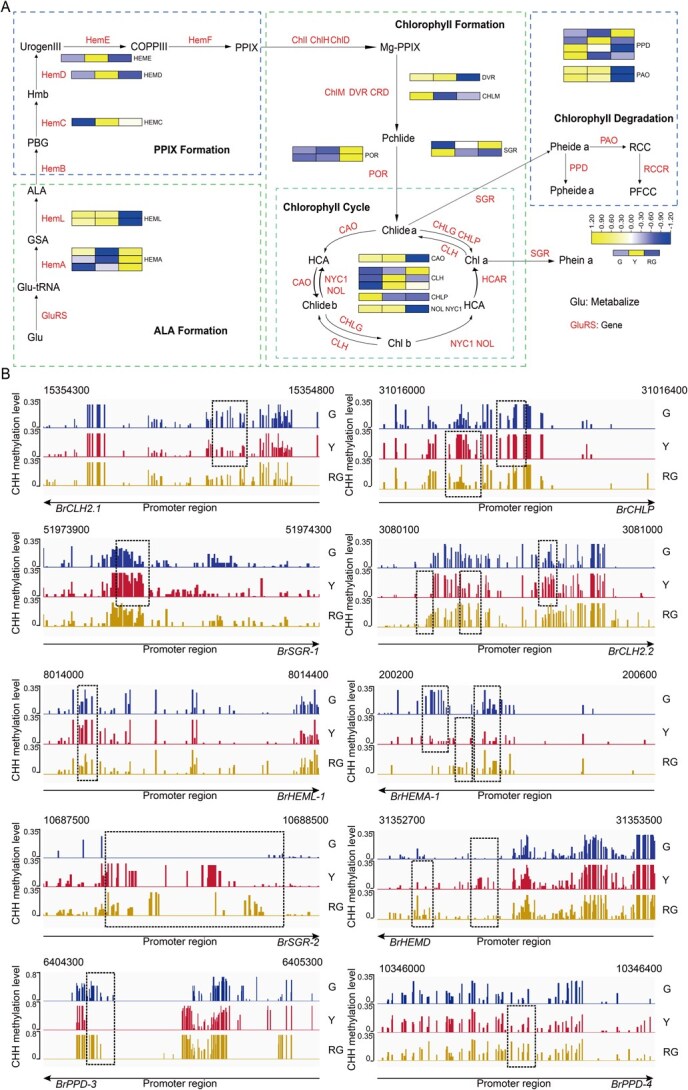
CHH methylation regulates genes involved in porphyrin and chlorophyll metabolism. (A) Heatmap of DMRs-associated DEGs related to porphyrin and chlorophyll metabolism in CHH. (B) The DNA methylation level in the CHH context. The black box represents DMR.

We further investigated the correlation linking promoter methylation dynamics with expression levels of chlorophyll biosynthesis/degradation genes. We found significant changes in CHH methylation in chlorophyll metabolism, and these methylation changes were associated with differential expression of chlorophyll metabolism-related genes (Fig. 5 and Fig. S20). Differential expression levels of *BrHemA*, *BrHemL*, *BrHemD*, *BrHemE*, *BrDVR*, *BrCHLM*, *BrCAO*, *BrCLH2*, *BrCHLP*, *BrNYC1/BrNOL*, *BrSGR*, *BrPPD*, and *BrPAO* were identified in Y/G or RG/Y. Differential methylation of *BrHemA*, *BrHemL*, *BrHemD*, *BrCLH2*, *BrCHLP*, *BrSGR*, and *BrPPD* was identified in Y/G or RG/Y. We estimated that CHH methylation in the promoter regions played a vital role in chlorophyll metabolism by regulating the expression of related genes in the inner leaves of wucai. Subsequent analyses revealed that *BrCLH2.1* exhibited increased transcription in the Y stage, concomitant with CHH hypomethylation in its promoter region. Conversely, *BrCLH2.2* showed reduced transcription in the RG stage, along with CHH hypermethylation in its promoter region (Fig. S20). Notably, *BrCLH2* expression levels were inversely correlated with promoter methylation levels, with the most significant changes observed. Additionally, *BrCLH2s* expression levels paralleled chlorophyll loss and recovery. Consequently, *BrCLH2s* were targeted as key candidate genes for further functional assays.

### Role of *BrCLH2* in chlorophyll degradation

**Figure 6:**
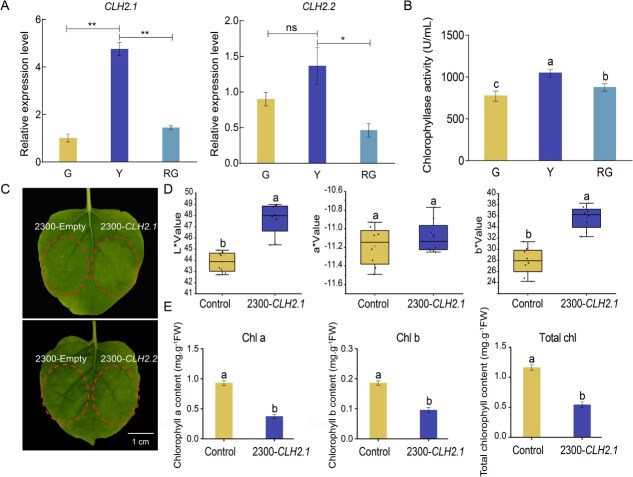
Functional verification of *BrCLH2* in chlorophyll degradation. (A) RT-qPCR analysis of *BrCLH2*. (B) Determination of chlorophyll enzyme activity at G, Y, and RG stages. (C) Transient expression analysis of *BrCLH2* in *N. benthamiana* leaves. (D) The change in color values L*, a*, and b* in *N. benthamiana* leaves transiently expressing *BrCLH2.1*. (E) The contents of Chl a, Chl b, and total Chl in *N. benthamiana* leaves transiently expressing *BrCLH2.1*. Data were derived from six biological replicates of each period. Error bars represent ±SD. Different letters indicate significant differences (*P* < 0.05). Different numbers of * above the bars indicate significant differences (^*^P < 0.05, ^**^P < 0.01).

Reverse transcription quantitative PCR (RT-qPCR) analysis revealed that *BrCLH2.1* expression was significantly up-regulated in the Y stage but down-regulated in the RG stage. In contrast, *BrCLH2.2* exhibited no significant change in the Y stage, while being significantly down-regulated in the RG stage (Fig. 6A). Chlorophyll enzyme activity assays demonstrated a marked increase during the Y stage, followed by a significant decrease in the RG stage (Fig. 6B).

Two differentially transcribed *BrCLH2* genes (*BrCLH2.1* and *BrCLH2.2*) were functionally characterized. Transient expression in *Nicotiana benthamiana* leaves revealed that only *BrCLH2.1* expressing leaves exhibited chlorophyll degradation phenotypes compared to wild-type controls (Fig. 6C). Colorimetric analysis showed significantly increased L* and b* values (Fig. 6D), concomitant with marked reductions in Chl a, Chl b, and total chlorophyll contents (Fig. 6E). These results demonstrate that *BrCLH2.1* mediates chlorophyll degradation in wucai inner leaves. RT-qPCR of chlorophyll degradation-related genes (*BrCLH2.1*, *BrCLH2.2*, *BrSGR-1*, *BrSGR-2*, *BrPAO-1*, *BrPPD-2*, *BrPPD-3*, *BrPPD-4*, and *BrPPD-5*) showed significant up-regulation following treatment with the methylation inhibitor 5-azacytidine (Fig. S21 and Table S26). These results indicate that DNA methylation plays a crucial regulatory role in the process of chlorophyll degradation.

To validate the technical validity of the WGBS data, we identified a DMR in the *BrCLH2.1* promoter (−448 to −423 bp). McrBC-PCR analysis targeting this DMR revealed DNA methylation levels consistent with the bisulfite sequencing results (Fig. S22). This correlative validation confirms the reliability of our methylome dataset.

## Discussion

### The chromosome-scale genome sequence of wucai and comparison with those of Chinese cabbage and pak choi

*Brassica* species, which belong to the Brassicaceae family, display abundant phenotypic variation and high economic value [3]. *Brassica rapa*, including Chinese cabbage, pak choi, turnip, and oil crops, exhibits extremely large morphological differences [7]. In this species, whole-genome sequencing of Chinese cabbage was first completed in 2011 [2], and assemblies of the yellow sarson (ssp. *trilocularis*) and pak choi (ssp. *chinensis*) genomes were also completed in turn [11, 12, 24]. In present study, we generated a chromosome-resolved genome assembly for *B*. *rapa* W7-2, integrating Oxford Nanopore long-read sequencing (N50 = 46.53 Mb) with Hi-C chromatin interaction scaffolding. The chromosome-scale genome assembly of *B. rapa* W7-2 provides crucial information for comparative genome studies on *B. rapa*. The assembled *B. rapa* W7-2 genome is 480.57 Mb in size, larger than that of Chinese cabbage (Chiifu), pak choi (NHCC001), and yellow sarson (Z1). The genomes of *Brassica* plants typically feature a large number of repetitive sequences. The percentage of repetitive sequences in the genome of *B. rapa* W7-2 was 55.08%, including 27.97% LTRs (Tables S13 and S14). The accumulation of numbers of TEs and LTRs may have driven the enlargement of the *B. rapa* W7-2 genome [25]. In our genome assembly, the BUSCO assessment identified 1614 complete orthologs, matching the count in the *B*. *rapa* NHCC001 genome and exceeding that in the *B*. *rapa* PC-fu and purple pak choi genomes [11, 13]. The higher proportion of complete BUSCOs compared to *B*. *rapa* NHCC001 indicates superior sequence integrity. However, the annotated BUSCO completeness (92.69%) falls below typical reference genome standards (>95%), likely due to limitations in gene prediction algorithms for identifying conserved genes. This annotation gap may lead to a minor underestimation of ancient gene duplication frequencies in WGT analyses. Nevertheless, validation through OrthoFinder-based core gene family assessment and MCScanX collinearity analysis confirms that the inferred WGT events are consistent with published *Brassica* reference genomes, supporting the robustness of our evolutionary conclusions [11].

Wucai is a cold-resistant vegetable that can overwinter in open air at temperatures above −10°C. Encouragingly, many unique genes and genes in families that underwent expansion during the evolution of *B. rapa* W7-2 are enriched in pathways related to cold tolerance, such as alpha linolenic acid metabolism, fatty acid biosynthesis, and biosynthesis of unsaturated fatty acids (Fig. 2B and Fig. S7). These genes may have made a certain contribution to the cold tolerance of wucai. This provides resources for the genetic improvement of cold tolerance traits in wucai.

### Global change and specific change in DNA methylation during temperature-dependent inner-leaf yellowing in wucai

At the adult stage, the inner leaves of W7-2 remained green under normal temperature conditions, but turned yellow upon exposure to low temperature. To further verify that leaf yellowing in W7-2 was induced by low temperature, we restored the plants to normal temperature after low-temperature-induced yellowing. The leaves subsequently regained their green color, confirming the reversible nature of this process (Fig. 3A and Fig. S9). In the Y stage, significant reductions in the contents of Chl a, Chl b, and total Chl were observed (Fig. S10). These findings suggest that changes in chlorophyll content are closely associated with the yellowing of inner leaves in response to low temperatures.

In recent years, DNA methylation has garnered significant attention due to its crucial role in various biological processes, including fruit ripening, flowering, and leaf senescence [26, 27]. For instance, during the development of lemon fruits, DNA methylation is essential in regulating citric acid metabolic pathways [28]. In the context of carnation flower senescence, the DNA demethylase *DcROS1* influences the aging process by modulating amino acid biosynthesis pathways [29]. Furthermore, DNA methylation has been shown to regulate the ripening processes in strawberries, citrus, peppers, and peaches [30–33]. However, there have been no reports on the DNA methylation level during the process of wucai color transformation. The present study revealed that DNA methylation plays an important role during inner-leaf yellowing of wucai. The DNA methylation level increased in the Y stage, only the CHH methylation levels decreased, and the CG and CHG methylation levels increased in the RG stage (Fig. 3C). This result suggests that low temperature changed the DNA methylation levels of the whole genome. Further analysis showed that the CHH methylation level was slightly decreased in the promoter and body regions of genes and flanking and body regions of TEs in the RG stage (Fig. 3D). Interestingly, the number of hyper-DMRs and hypo-DMRs gradually decreased in the Y/G and RG/Y comparisons, but the number of hypo-DMRs in RG/Y was increased significantly in the CHG and CHH contexts, particularly in the CHH context (Fig. S13). A comparative analysis indicated that DMRs of the three contexts in the Y stage showed higher methylation levels than those in the G stage, but only the CHH context in the RG stage displayed lower methylation levels than in the Y stage (Fig. 4A). The distribution of DMRs was analyzed, and most hyper-DMRs and hypo-DMRs were distributed in the promoter region in the CHH context in RG/Y (Fig. S14). These results show that CHH methylation plays a key role in the low-temperature acclimation of wucai.

The dynamic equilibrium of cytosine methylation is maintained through antagonistic regulation by DNA methyltransferases (writers) and DNA demethylases (erasers). Thale cress (*A. thaliana*) is a model species for plant genetics. METHYLTRANSFERASE1 (MET1) maintains CG methylation, while CHG methylation is maintained by CHROMOMETHYLASE 3 (CMT3) during DNA replication. In particular, CHROMOMETHYLASE 2 (CMT2) and DOMAINS REARRANGED METHYLTRANSFERASE2 (DRM2) act mainly in the CHH context [34–36]. In our study, the transcript levels of *BrCMT2* and *BrDRM2* were consistent with the change in DNA methylation levels accompanying the transition from low-temperature colors to room-temperature green (Fig. S15). Therefore, we speculate that wucai DNA methylation status is regulated by *BrCMT2* and *BrDRM2.*

DNA methylation serves as a key epigenetic mechanism modulating transcriptional regulation [37]. It regulates the level of DNA methylation through methylation and demethylation and then regulates the expression of related genes. In the present study, there were differences in the expression and methylation levels of chlorophyll metabolism-related genes, such as *BrHemA*, *BrHemL*, *BrHemD*, *BrCLH2*, *BrCHLP*, *BrSGR*, and *BrPPD* in the Y/G and RG/Y comparisons (Fig. 5 and Fig. S20)*.* These genes exhibited CHH methylation in the promoter region. The results suggest that CHH methylation in the promoter regions plays a role in the regulation of porphyrin and chlorophyll metabolism-related gene expression in wucai. RT-qPCR analysis of chlorophyll degradation-related genes revealed significant up-regulation of *BrCLH2.1*, *BrCLH2.2*, *BrSGR-1*, *BrSGR-2*, *BrPAO-1*, *BrPPD-2*, *BrPPD-3*, *BrPPD-4*, and *BrPPD-5* following 5-azacytidine treatment (Fig. S21). Conversely, *BrCMT2* and *BrDRM2* exhibited significant down-regulation upon inhibitor exposure (Fig. 4C). These results support the regulatory role of DNA methylation in chlorophyll degradation.

Furthermore, *BrCLH2.1* and *BrCLH2.2* were identified through transcriptome and DNA methylome analyses as DEGs within porphyrin and chlorophyll metabolic pathways. Transient expression analysis of *BrCLH2.1* and *BrCLH2.2* genes in *N. benthamiana* showed that only *BrCLH2.1*-expressing leaves exhibited chlorophyll degradation phenotypes (Fig. 6C), indicating a critical role for *BrCLH2.1* in chlorophyll degradation in the inner leaves of wucai. DNA methylation regulates biological processes via direct modulation of specific gene expression [38, 39], transcription factor-mediated mechanisms [40, 41], or combining the two ways [42]. As it was uncertain whether changes in the *BrCLH2.1* expression level were regulated by DNA methylation or transcriptional regulation, more experiments and analyses are needed in future studies.

## Materials and methods

A full description can be found in the Supplemental Materials and Methods, including genome sequencing, assembly, and chromosome construction, gene prediction and functional annotation, gene family and phylogenetic analysis, chromosome collinearity, and whole-genome duplication analysis. The wucai cultivar ‘W7-2’ from Anhui Agricultural University was chosen for the sequencing and assembly of the reference genome. Leaf color values were estimated by a DS-700D chroma meter (Hangzhou CHNSpec Technology Co., Ltd) on the surface of the leaf every 3 days after planting. The contents of photosynthetic pigments (Chl a and Chl b) were measured using the traditional method of Arnon. RNA-Seq and WGBS-Seq were performed by BGI (https://www.bgi.com/global/) according to their standard analysis procedure.

## Consent for publication

All authors approve the manuscript and consent to publication of the work.

## Supplementary Material

Web_Material_uhaf231

## Data Availability

The raw sequence data reported in this paper have been deposited in the National Genomics Data Center (GSA: CRA012429, CRA012488, and CRA012487).
