# Supplementary material for: The wucai genome and DNA methylation regulation on the inner leaves’ yellowing response to low temperature
Source: Hortic Res. 2025 Sep 2;12(12):uhaf231. doi: 10.1093/hr/uhaf231 (PMC12680381; doi:10.1093/hr/uhaf231)
Supplement: Web_Material_uhaf231 [file web_material_uhaf231.zip › Revised supplementary files.pdf]

Supplemental files - The wucai genome and DNA methylation regulation on the inner leaves yellowish response to low temperature

Supplemental Materials and Methods

Section S1- Materials and genome sequencing
Section S2- Genome assembly and chromosome construction
Section S3- Gene prediction and functional annotation
Section S4- Functional annotation of protein-coding genes
Section S5- Gene families and phylogenetic analysis
Section S6- Chromosome collinearity and whole-genome duplication analysis
Section S7- Experimental treatments
Section S8- Measurement of color values
Section S9- Measurement of chlorophyll content
Section S10- RNA sequencing and analysis
Section S11- Whole-genome bisulfite sequencing and analysis
Section S12-Transient expression analysis of tobacco leaves
Section S13-Real-time quantitative PCR (RT-qPCR)
Section S14-McrBC-PCR

Supplemental Materials and Methods

Section S1- Materials and genome sequencing

‘W7-2’ is a typical inbred line with yellowish inner leaves at the adult stage in autumn and winter and is widely used as a parent to develop hybrid varietie[1]. Fresh leaf tissues were collected and immediately frozen in liquid nitrogen to extract genomic DNA and RNA. The CTAB method was used to extract high-quality genomic DNA from leaves[2]. We used single-molecule real-time sequencing (SMRT) technology, the third-generation sequencing technology of Pacific Biosciences (Menlo Park, CA, USA), to sequence the W7-2 genome, and reads with a total length equivalent to ~1081× genome coverage were obtained. High-quality data were obtained and screened on the Illumina HiSeq sequencing platform with Q20 ≥97.58%. Transcriptomes of different W7-2 tissues were sequenced and used for genome assembly. All transcriptome libraries were sequenced using the Illumina platform, generating paired-end reads.

Section S2- Genome assembly and chromosome construction

For genome survey analysis, a short paired-end Illumina DNA library with a 350 bp insert size (131.9× coverage) was sequenced on an Illumina HiSeq 2500 sequencer. For PacBio sequencing, 50 µg of high-molecular-weight genomic DNA was prepared to generate two standard cell libraries with 20 kb insertions. The extracted DNA was sequenced on the PacBio Sequel II platform (Pacific Biosciences, Menlo Park, CA, USA) with the CCS model and two SMRT cells. The resulting BAM files were processed using the pbccs program (http://ccs.how/) with default parameters, producing 508.2 Gb (~1081×) of HiFi reads. We further used the Hifiasm[3] assembler (v.0.15.4) to assemble these HiFi reads with default parameters and purge_haplotigs (v1.1.1) to extract phased contigs. For Hi-C sequencing and scaffolding, a Hi-C library was created from tender leaves of *B. rapa* wucai. In brief, the leaves were fixed with formaldehyde and lysed, and the cross-linked DNA was then digested with MboI overnight. Sticky ends were biotinylated and proximity-ligated to form chimeric junctions, which were physically sheared to and enriched for sizes of 500–700 bp. Chimeric fragments representing the original cross-linked long-distance physical interactions were then processed into paired-end sequencing libraries and 346 million 150-bp paired-end reads, which were sequenced on the Illumina platform. A total of 4.90 Gb of Hi-C reads (Q20≥97.85%) were mapped to the initially phased contigs by using BWA-MEM[4], and uniquely mapped reads were extracted using Juncer9 (v1.5.6). We then used 3d-dna[5] to divide, sequence and orient the genome sequences and evaluate the assembly results. The genome assembled by HI-C into chromosomes was cut into 1500 kb bins of equal length, and then the numbers of Hi-C read pairs between any two bins were used as the intensity signal of the interaction between the two bins to create a heatmap. Based on the single-copy homologous plant-specific database, the completeness of the genome assembly was assessed using BUSCO[6].

Section S3- Gene prediction and functional annotation

Genome-wide repetitive sequences were annotated by de novo and homology-based approaches. Based on the Repbase v 21.01 database (www.girinst.org/repbase)[7], homolog prediction was performed using RepeatMasker v 4.1.2 and RepeatProteinMask v 4.1.2 (http://www.repeatmasker.org/cgibin/RepeatProteinMaskRequest) to identify sequences similar to known repetitive sequences. De novo prediction was performed using self-sequences or transposon structure characteristics to construct a de novo repetitive sequence database

through RepeatModeler (http://www.repeatmasker.org/RepeatModeler/) and LTRharvest v1.6.1 (http://github.com/genometools/genometools)[8,9] and then to detect repetitive sequences. Additionally, Tandem Repeats Finder v4.09 (http://tandem.bu.edu/trf/trf.html)[10] was used to detect tandem repeats in the genome.

We employed homology prediction, combined with RNA data, to detect gene structure using the genomes of *A. thaliana*, *B. napus*, *B. oleracea*, *B. rapa* and *O. sativa*. GeMoMa v1.7.1 (http://www.jstacs.de/index.php/GeMoMa) was used for gene structure prediction[11]. Finally, we filtered these genes with the criterion of coding sequence (CDS) length>150 kb and exon number >1 to obtain the final results.

In this project, the full-length mRNA sequences of five different wucai tissues, namely, roots, stems, leaves, flowers and seeds, were used for auxiliary annotation. We constructed a SMRTbell library, and sequencing data were processed through reads of insert (ROI), full-length transcript classification, clustering and polishing to consensus reads. The consensus reads were then mapped to the reference genome for fusion gene detection, APA site detection, isoform classification, functional annotation, coding capacity prediction and AS gene detection. We mapped the polished isoforms to the reference genome and detected fusion genes using the fusion_finder.py program from official TOFU analysis toolkits. The Cuffcompare program of cufflinks software was used to compare the deduplicated isoforms with the known gene annotations and classify the isoforms. To detect AS events, ASprofile V b-1.0.4 (http://ccb.jhu.edu/software/ASprofile) was performed on the sample.

Section S4- Functional annotation of protein-coding genes

Predicted genes were subjected to functional annotation by performing a BLASTP homolog search against the Blast v2.2.31 database[12]. Comprehensive annotation was also carried out using InterProScan (v5.31–70.0)[13,14], which includes motif/domain prediction, functional classification, protein family identification, transmembrane topology inference, signal peptide prediction and GO annotation. In addition, the SwissProt (https://www.uniprot.org/), TrEMBL (https://www.ebi.ac.uk/uniprot/), KEGG (https://www.genome.jp/kegg/), InterPro (https://www.ebi.ac.uk/interpro/), NR, KOG and GO databases were also used to generate gene functional information.

Section S5- Gene families and phylogenetic analysis

Gene families/clusters in the genomes of *B. rapa* W7-2 and other species were identified using the OrthoFinder package (v2.3.11)[15]. The protein sequences of 154 single-copy orthologs from 15 species were concatenated for species tree construction. Gene family expansion and contraction in the *B. rapa* W7-2 genome were determined by comparison with genes in 14 other species using Computational Analysis of Gene Family Evolution (CAFE) (v4.2.1)[16]. *O. sativa* was designated as the outgroup. To determine divergence times based on the phylogenetic tree, MCMCTree implemented in the PAML program was used[17]. Divergence time calibration information was obtained from the TimeTree database (http://www.timetree.org/).

Section S6- Chromosome collinearity and whole-genome duplication analysis

Homologous genes within or between the genomes of two species were identified using the BLASTP program (*E*-value <1e-5). MCScanX[18] was used to find the collinear regions, and JCVI (v0.8.12) (https://github.com/tanghaibao/jcvi)[19] software was used to analyze the collinearity between species. *Ks* was calculated using the codeml program of PAML. The distribution of *Ks* density was determined using WGDI[20]. The curve of the *Ks* density

distribution wasdrawn using Kspeaks.

Section S7- Experimental treatments

Leaves of W7-2 turn yellow during the growing period when gradually experiencing low temperatures in autumn and winter. In this study, W7-2 seedlings were planted in a greenhouse at 26 ± 2°C (day) and 20 ± 2°C (night) with 300 µmol $m^{-2}s^{-1}$ light intensity and 70−80% relative humidity. Seedlings with 7-8 leaves were transplanted to a low-temperature growth chamber at 6 ± 2°C (day) and -1 ± 2°C (night) with 300 µmol $m^{-2}s^{-1}$ light intensity and 70−80% relative humidity for 12 days. Then, the temperature was set to 26 ± 2°C (day) and 20 ± 2°C (night) with 300 µmol $m^{-2}s^{-1}$ light intensity and 70−80% relative humidity for 12 days. After transfer to the growth chamber, the third fully expanded young leaf was collected from the center every 3 days. Samples were immediately frozen in liquid nitrogen and stored at −80°C for physiological and biochemical measurements[21].

Section S8- Measurement of color values

Leaf color values were estimated by a DS-700D chroma meter (Hangzhou CHNSpec Technology Co., Ltd.) on the surface of the leaf every 3 days after planting. The color data were analyzed by the CIELAB color coordinate system[1]. There are three representative color values: L*, a*, and b*. L* represents brightness (black and white), a* represents red and green, and b* represents yellow and blue.

Section S9- Measurement of chlorophyll content

The contents of photosynthetic pigments (Chl a and Chl b) were measured using the traditional method of Arnon. The absorbance at wavelengths of 470, 646, and 663 nm was analyzed by a TU1950 UV−vis spectrophotometer (PER-SEE, Beijing, China). The Chl a (mg/g) and Chl b (mg/g) contents were calculated as follows[21]:

Chl a mg/L = 12.21 ×A663 − 2.81 ×A646;

Chl b mg/L = 20.13 ×A646 − 5.03 ×A663;

Chl a mg/g = Chl a mg/L ×V (L)/Mfresh

Chl b mg/g = Chl b mg/L ×V (L)/Mfresh (g);

Section S10- RNA sequencing and analysis

The total RNA of 9 samples from three periods was extracted by using the CTAB method, and RNA-seq libraries were constructed using a Qubit® ssDNA Assay kit. Then, the libraries were sequenced on the DNBSEQ platform (MGISEQ-2000RS and DNBSEQ-T7RS). The raw data obtained by sequencing were called raw reads. To ensure the reliability of the results, SOAPnuke was used to remove raw reads with low quality, splice contamination, and excessive unknown base (N) content to generate clean reads. After obtaining clean reads, we aligned the clean reads to the reference genome sequence using HISAT. Clean reads were aligned to the *B. rapa* W7-2 reference gene sequences using Bowtie2 to obtain the alignment results. Fragments per kilobase of transcript per million mapped reads (FPKM) values were calculated to represent the expression level of mRNAs[22]. DEseq2 was used to detect the DEGs. Genes with false discovery rate (FDR)<0.05 and |log2(fold change)|>1 were defined as DEGs. Then, GO and KEGG analyses were performed for the DEGs.

Section S11- Whole-genome bisulfite sequencing and analysis

DNA was extracted from W7-2 leaves at different periods using the CTAB method. For normal WGBS library construction, the DNA was fragmented by sonication using a Bioruptor (Diagenode, Belgium) to a mean size of approximately 250 bp, followed by blunt-ending, dA

addition to the 3'-end, and adaptor ligation (in this case, ligation of methylated adaptors to protect from bisulfite conversion), essentially according to the manufacturer's instructions. Ligated DNA was bisulfite converted using the EZ DNA Methylation-Gold Kit (ZYMO). Different-insert-size fragments were excised from the same lane of a 2% TAE agarose gel. Products were purified by using a QIAquick Gel Extraction kit (Qiagen) and amplified by PCR. Finally, sequencing was performed using the HighSeq4000 platform.

The reads were filtered via BGI programs. After filtering, the clean reads were mapped to the wucai genome using BSMAP, duplicate reads were removed, and the mapping results were merged according to each library. We calculated the mapping rate and bisulfite conversion rate of each sample. The methylation level was determined by dividing the number of reads covering each mC by the total reads covering that cytosine, which was also equal to the mC/C ratio at each reference cytosine. We calculated the degree of difference for a methylcytosine (mCG, mCHG, mCHH) by comparing its methylation level between two samples while comparing the methylation level of DMRs in different samples by CIRCOS. DMRs were identified by $P < 0.05$ (ANOVA test) and absolute methylation difference over 0.1.

Section S12-Transient expression analysis of tobacco leaves

Preparation of Activation buffer: MES and MgCl2 are mixed in a ratio of 5:1 and sterilized at high temperature. The transformed Agrobacterium tumefaciens was shaken overnight in a shaking bed at 28°C, and the appropriate OD value of the bacterial solution was 0.8-1.0. Centrifuge at 4000 rpm for 8 min, remove the supernatant, suspend with 3 ml ddH2O, centrifuge at 4000 rpm for 8min, and then suspend with Activation buffer. Centrifuge at 4000 rpm for 8 min. OD values were determined with Activation buffer as control, and the OD values were adjusted from 0.8 to 1.0 with Activation buffer. AS was added to the bacterial solution at the ratio of AS= 1000:1. After static dark culture at 28°C for 3 h, *N. benthamiana* leaves was injected. After injection, it was treated in the dark for 1 day and cultured under light for about 2 days. In addition, leaf phenotype was observed, leaf color value, and chlorophyll content were measured after tobacco injection.

Section S13-Real-time quantitative PCR (RT-qPCR)

According to the nucleotide sequences of target genes in the genome of wucai, fluorescent primers of target genes were designed using Oligo7 software (Table S23) to detect the expression levels of target genes. Actin was used as the internal reference gene, and the primer sequences were sent to General Biological Company for synthesis. The qRT-PCR system was as follows: 2 × PerfectStart® Green qPCR SuperMix 10 µL, F: 0.4 µL, R: 0.4 µL, cDNA 1 µL, DEPC 8.2 µL. qRT-PCR procedures were as follows: 95°C 30 s, 95°C 5 s, 60°C 15 s, 72°C 10 s, image acquisition, cycle 40 times, dissolution curve 58°C to 95°C 5 s, image acquisition.

Section S14-McrBC-PCR

The extracted DNA was digested with McrBC endo-nuclide (TaKaRa) at 37°C for 3 hours. The undigested DNA was used as the control for PCR reaction. The PCR procedure was 95°C for 5 min, 95°C for 30 s, 60°C for 30 s, 72°C for 2 min. 72°C for 5 min. After the reaction, the PCR products were identified by 1% agarose gel electrophoresis.

References

1. Xie, S.L. *et al.* Comparative proteomic analysis reveals that chlorophyll metabolism contributes

to leaf color changes in Wucai (*Brassica campestris* L.) responding to cold acclimation. *Journal of Proteome Research* **18**, 2478-2492 http://dx.doi.org/10.1021/acs.jproteome.9b00016 (2019).
2. Allen, G.C., Flores-Vergara, M.A., Krasnyanski, S., Kumar, S. & Thompson, W.F. A modified protocol for rapid DNA isolation from plant tissues using cetyltrimethylammonium bromide. *Nature Protocols* **1**, 2320-2325 http://dx.doi.org/10.1038/nprot.2006.384 (2006).
3. Cheng, H.Y., Concepcion, G.T., Feng, X.W., Zhang, H.W. & Li, H. Haplotype-resolved de novo assembly using phased assembly graphs with hifiasm. *Nature Methods* **18**, 170-175 http://dx.doi.org/10.1038/s41592-020-01056-5 (2021).
4. Li, H. & Durbin, R. Fast and accurate short read alignment with Burrows-Wheeler transform. *Bioinformatics* **25**, 1754-1760 http://dx.doi.org/10.1093/bioinformatics/btp324 (2009).
5. Dudchenko, O. *et al.* De novo assembly of the *Aedes aegypti* genome using Hi-C yields chromosome-length scaffolds. *Science* **356**, 92-95 http://dx.doi.org/10.1126/science.aal3327 (2017).
6. Simao, F.A., Waterhouse, R.M., Ioannidis, P., Kriventseva, E.V. & Zdobnov, E.M. BUSCO: assessing genome assembly and annotation completeness with single-copy orthologs. *Bioinformatics* **31**, 3210-3212 http://dx.doi.org/10.1093/bioinformatics/btv351 (2015).
7. Bao, W.D., Kojima, K.K. & Kohany, O. Repbase Update, a database of repetitive elements in eukaryotic genomes. *Mobile DNA* **6**, 11 http://dx.doi.org/10.1186/s13100-015-0041-9 (2015).
8. Ellinghaus, D., Kurtz, S. & Willhoeft, U. *LTRharvest*, an efficient and flexible software for de novo detection of LTR retrotransposons. *BMC Bioinformatics* **9**, 18 http://dx.doi.org/10.1186/1471-2105-9-18 (2008).
9. Price, A.L., Jones, N.C. & Pevzner, P.A. *De novo* identification of repeat families in large genomes. *Bioinformatics* **21**, I351-I358 http://dx.doi.org/10.1093/bioinformatics/bti1018 (2005).
10. Benson, G. Tandem repeats finder: a program to analyze DNA sequences. *Nucleic acids research* **27**, 573-580 http://dx.doi.org/10.1093/nar/27.2.573 (1999).
11. Keilwagen, J., Hartung, F. & Grau, J. GeMoMa: homology-based gene prediction utilizing intron position conservation and RNA-seq data. *Methods in molecular biology (Clifton, N.J.)* **1962**, 161-177 http://dx.doi.org/10.1007/978-1-4939-9173-0_9 (2019).
12. Altschul, S.F., Gish, W., Miller, W., Myers, E.W. & Lipman, D.J. Basic local alignment search tool. *Journal of molecular biology* **215**, 403-410 http://dx.doi.org/10.1016/s0022-2836(05)80360-2 (1990).
13. Boeckmann, B. *et al.* The SWISS-PROT protein knowledgebase and its supplement TrEMBL in 2003. *Nucleic Acids Research* **31**, 365-370 http://dx.doi.org/10.1093/nar/gkg095 (2003).
14. Zdobnov, E.M. & Apweiler, R. InterProScan - an integration platform for the signature-recognition methods in InterPro. *Bioinformatics* **17**, 847-848 http://dx.doi.org/10.1093/bioinformatics/17.9.847 (2001).
15. Emms, D.M. & Kelly, S. OrthoFinder: solving fundamental biases in whole genome comparisons dramatically improves orthogroup inference accuracy. *Genome Biology* **16**, 157 http://dx.doi.org/10.1186/s13059-015-0721-2 (2015).
16. De Bie, T., Cristianini, N., Demuth, J.P. & Hahn, M.W. CAFE: a computational tool for the study of gene family evolution. *Bioinformatics* **22**, 1269-1271 http://dx.doi.org/10.1093/bioinformatics/btl097 (2006).
17. Yang, Z.H. PAML 4: Phylogenetic analysis by maximum likelihood. *Molecular Biology and*

Evolution **24**, 1586-1591 http://dx.doi.org/10.1093/molbev/msm088 (2007).

18. Wang, Y.P. *et al. MCScanX*: a toolkit for detection and evolutionary analysis of gene synteny and collinearity. *Nucleic Acids Research* **40**, e49 http://dx.doi.org/10.1093/nar/gkr1293 (2012).
19. Goll, J. *et al.* METAREP: JCVI metagenomics reports-an open source tool for high-performance comparative metagenomics. *Bioinformatics* **26**, 2631-2632 http://dx.doi.org/10.1093/bioinformatics/btq455 (2010).
20. Sun, P.C. *et al.* WGDI: A user-friendly toolkit for evolutionary analyses of whole-genome duplications and ancestral karyotypes. *Molecular Plant* **15**, 1841-1851 http://dx.doi.org/10.1016/j.molp.2022.10.018 (2022).
21. Yuan, L.Y. *et al.* Comparative transcriptome analysis reveals that chlorophyll metabolism contributes to leaf color changes in wucai (*Brassica campestris* L.) in response to cold. *BMC Plant Biology* **21**, 438 http://dx.doi.org/10.1186/s12870-021-03218-9 (2021).
22. Lyu, Z.R. *et al.* Transcriptome and DNA methylome provide insights into the molecular regulation of drought stress in sea buckthorn. *Genomics* **114**, 110345 http://dx.doi.org/10.1016/j.ygeno.2022.110345 (2022).

Abbreviations

BUSCO: benchmark universal single copy homologous sequences

TE: transposable element

KEGG: Kyoto Encyclopedia of Genes and Genomes

GO: Gene Ontology

AS: alternative splicing

LINEs: long interspersed nuclear elements

SINEs: short interspersed nuclear elements

LTRs: long terminal repeats

Ks: synonymous substitution rates

SV: Structural variation

G: Low temperature before color change

Y: low temperature after color change

RG: normal temperature after color change

WGBS: whole genome bisulfite sequencing

DMGs: Differentially methylated genes

DMR: Differentially methylated regions

DEGs: differentially expressed genes

MET1: METHYLTRANSFERASE 1

CMT2: CHROMOMETHYLASE 2

CMT3: CHROMOMETHYLASE 3

DRM2 DOMAINS REARRANGED METHYLTRANSFERASE 2

DML2: Demeter-like protein 2

DML3: Demeter-like protein 3

GluRS: Glutamyl/glutaminyl-tRNA synthetase

HEMA: glutamyl-tRNA reductase

HEML: glutamate-1-semialdehyde 2,1-aminomutase

HEMB: porphobilinogen synthase

HEMC: hydroxymethylbilane synthase

HEMD: uroporphyrinogen-III synthase

HEME: uroporphyrinogen decarboxylase

HEMF: coproporphyrinogen III oxidase

CHLH: magnesium chelatase subunit H

CHLI: magnesium chelatase subunit I

CHLD: magnesium chelatase subunit D

CHLM: Magnesium-protoporphyrin IX methyltransferase

DVR: 3,8-Divinylprotochlorophyllide a 8-vinyl reductase

CRD: Chloroplast Relocation Defective

POR: NADPH-protochlorophyllide oxidoreductase

CAO: chlorophyllide a oxygenase

CHLG: chlorophyll/bacteriochlorophyll a synthase

CLH: chlorophyllase

CHLP: geranylgeranyl reductase

HCAR: 7-hydroxymethyl chlorophyll a reductase

NOL: non-yellow coloring-like
NYC1: non-yellow coloring
SGR: Stay-Green Rice
PAO: pheophorbide a oxygenase
PPD: pheophorbidase
RCCR: red chlorophyll catabolite reductase

Supplemental Figures

Fig. S1 Distribution of 25-mer frequency in the garlic genome.
Fig. S2 Heatmap showing Hi-C interactions in *B. rapa* W7-2 genome.
Fig. S3 Annotation information for transcripts in multiple databases.
Fig. S4 Phylogenetic tree of 19 sequenced species genomes.
Fig. S5 Venn diagram of the common and unique gene families among different brassica genomes.
Fig. S6 Distribution of genes in different species.
Fig. S7 Significant KEGG pathways enriched in specific genes of *B. rapa* W7-2.
Fig. S8 Synteny analysis based on the comparison of the 10 chromosomes assembled for *B. rapa* W7-2 and other *B. rapa* sepecies.
Fig. S9 Changes in the color L*value, a*value, and b*value in G, Y and RG (B).
Fig. S10 The Chl content during the three periods G, Y, and RG.
Fig. S11 Proportions of mC in three contexts (CG, CHG, and CHH) in wucai.
Figure S12 Average methylation level of mC, mCG, mCHG, and mCHH in some genomic elements.
Fig. S13 Numbers of hyper-differentially methylated regions (hyper-DMRs) and hypo-DMRs associated genes and promoters in Y/G and RG/Y.
Fig. S14 Distribution of hyper-DMRs and hypo-DMRs in Y/G and RG/Y under mCG, mCHG and mCHH contexts.
Fig. S15 Phylogenetic analysis and expression of DNA methyltransferase genes and DNA demethylasegenes (DML) in G, Y, and RG.
Fig. S16 The number of DEGs was obtained by comparing Y/G and RG/Y.
Fig. S17 Venn diagram of the resulting DEGs for Y/G and RG/Y comparisons.
Fig. S18 Hierarchical clustering of DEGs co-expressed between Y/G and RG/Y.
Fig. S19 (A)The number of DMRs and DMRs-associated DEGs in Y/G and RG/Y. (B) The Venn diagram shows the number of DMRs and DMRs-associated DEGs in CHH.
Fig. S20 Correlation analysis between DNA methylation levels and expression levels in chlorophyll metabolism during inner-leaf yellowing in wucai.
Fig. S21 The expression of chlorophyll degradation-related genes in mock and 100-Aza treated wucai inner-leaf.
Fig. S22 Analysis of promoter methylation levels of *BrCLH2.1* in three stages.

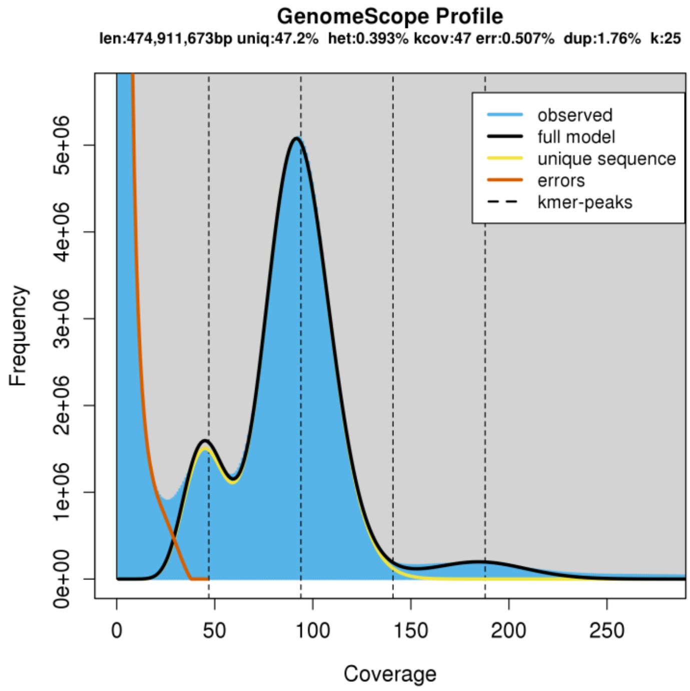

Fig. S1 Distribution of 25-mer frequency in the garlic genome. The abscissa is the 25 *k*-mer depth (coverage), and the ordinate is the 25 *k*-mer frequency at this depth.

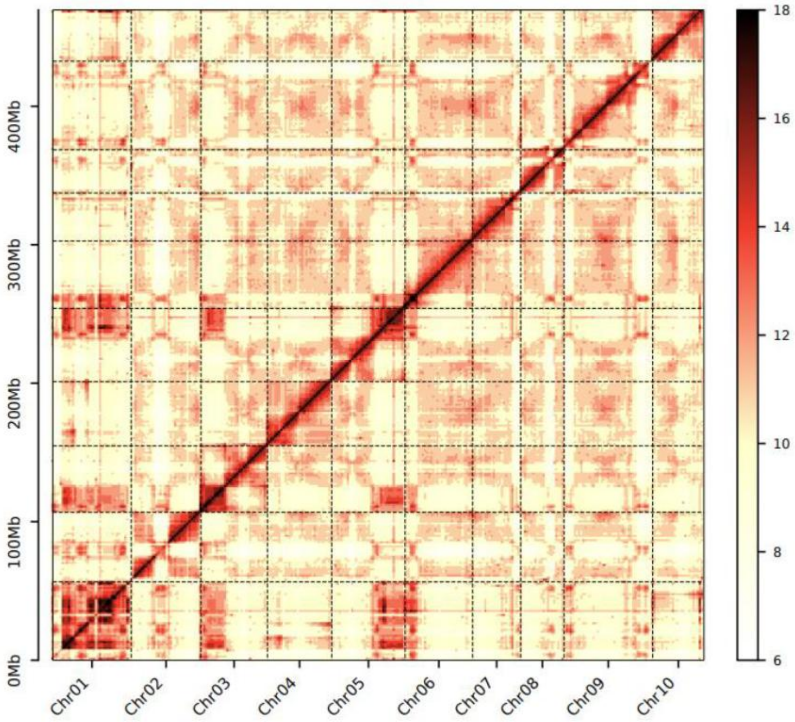

Fig. S2 Heatmap showing Hi-C interactions in *B*. *rapa* W7-2 genome. Each heat map shows a normalized contact matrix, with strong contacts in red and weak contacts in yellow.

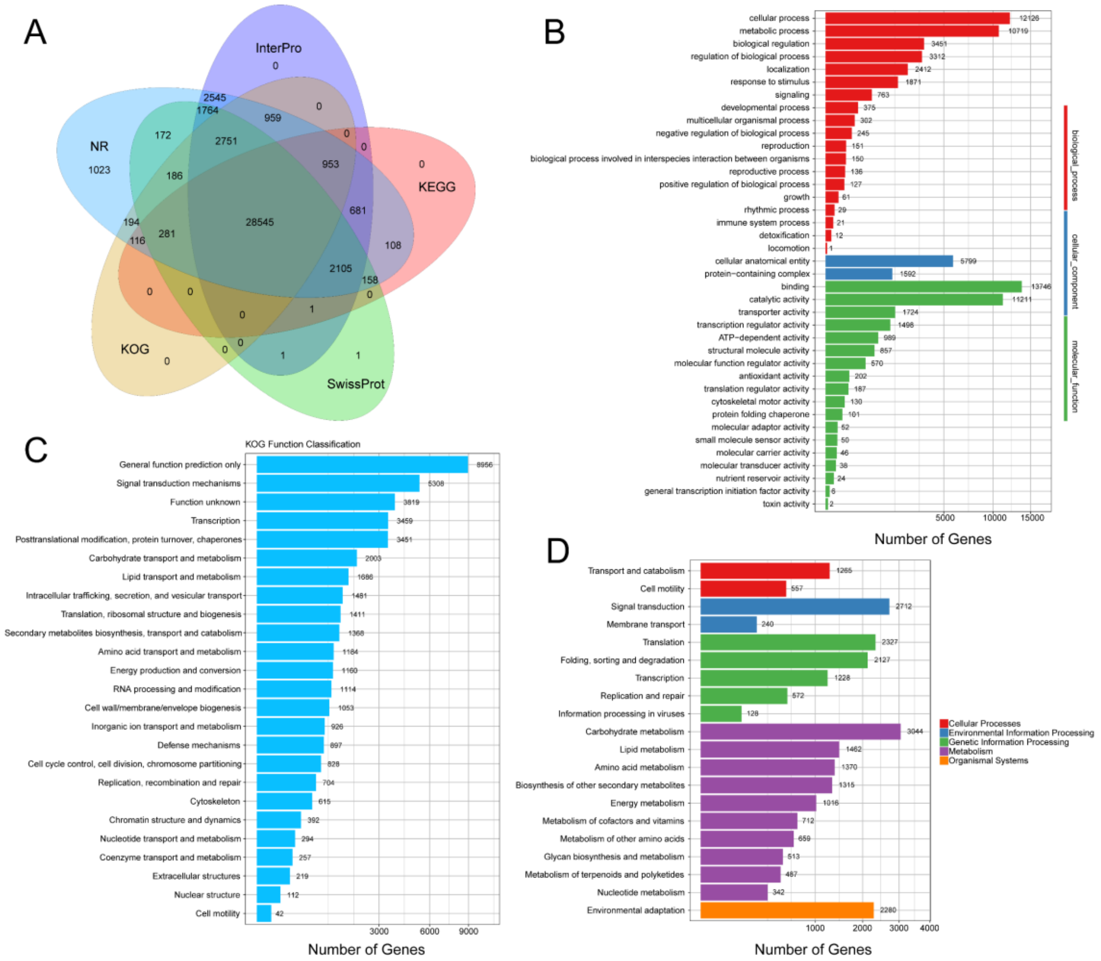

Fig. S3 Annotation information for transcripts in multiple databases.

A. Annotation distribution of transcripts in five databases: NR, SwissProt, KEGG, KOG, and InterPro. B. Functional classification of unigenes in the GO database. C. Functional classification of unigenes in the KOG database. D. Functional classification of unigenes in the KEGG database.

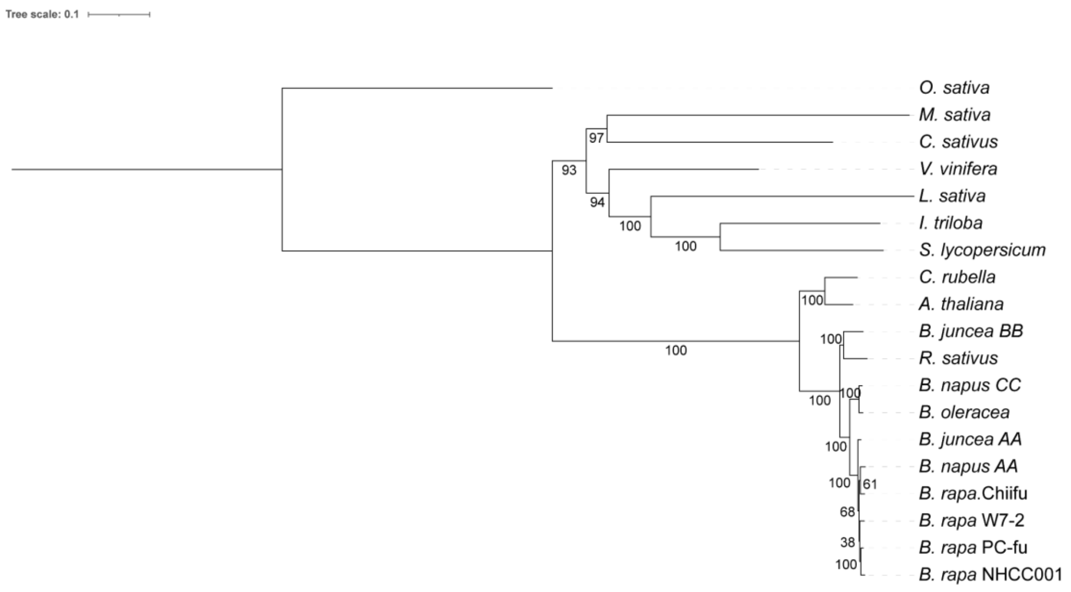

Fig. S4 Phylogenetic tree of 19 sequenced species genomes.

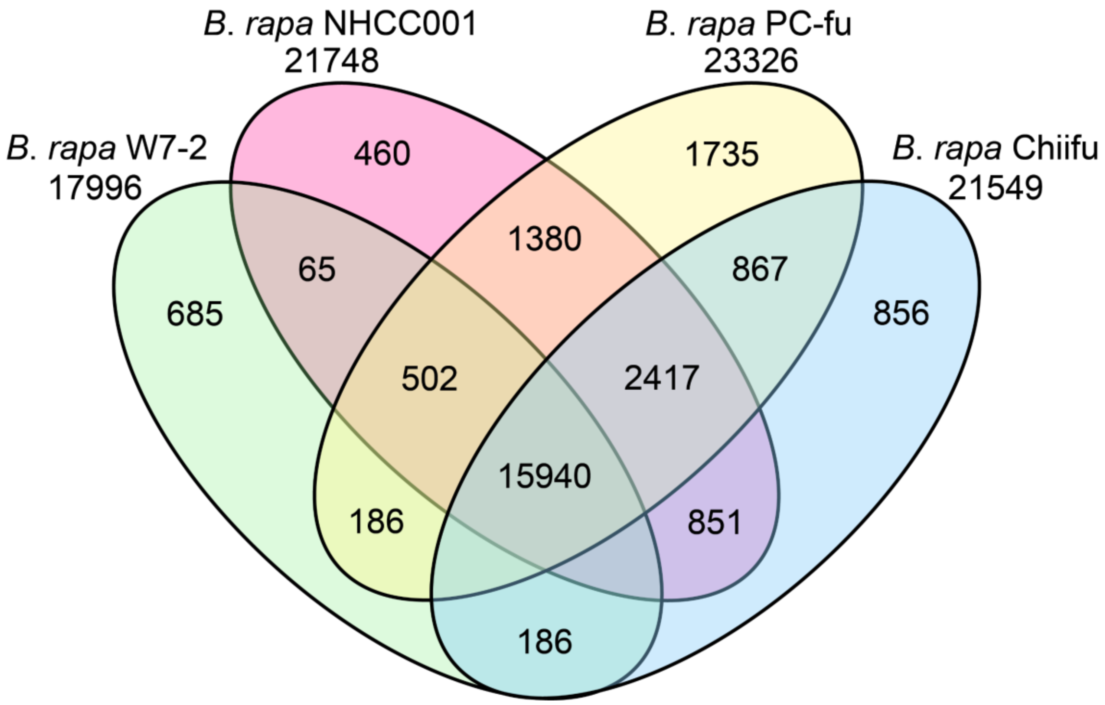

Fig. S5 Venn diagram of the common and unique gene families among different brassica genomes.

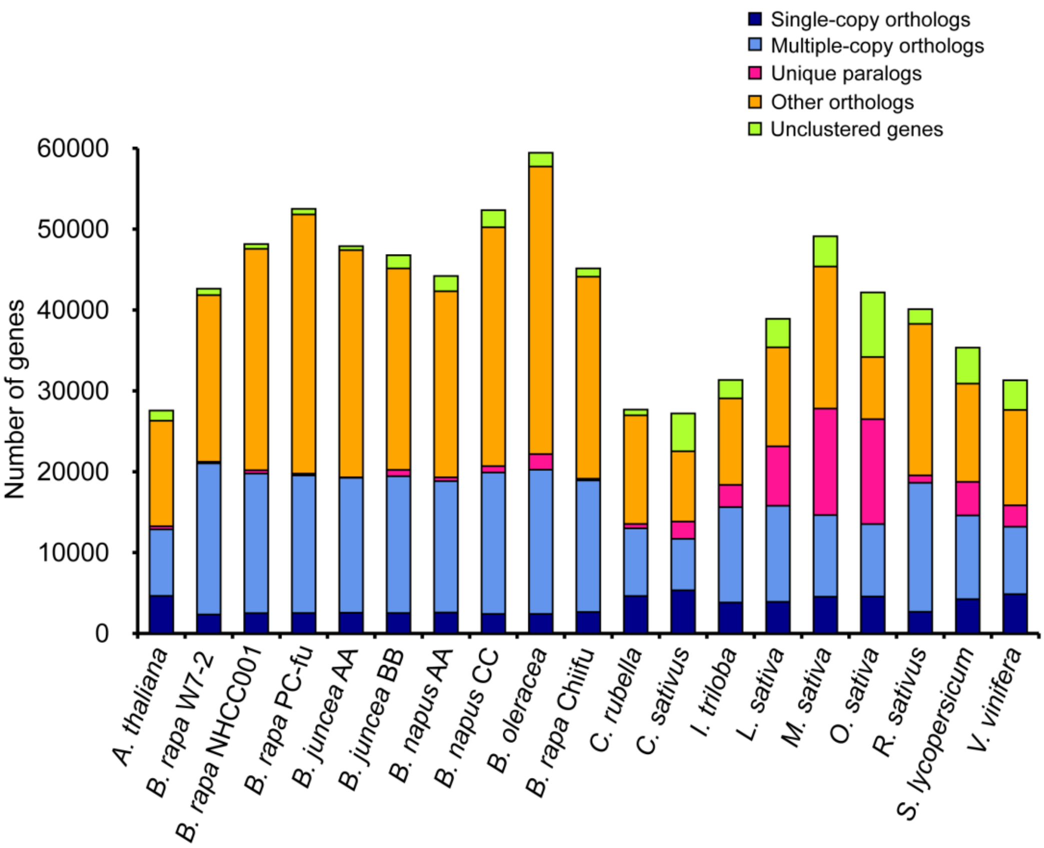

Fig. S6 Distribution of genes in different species.

The horizontal axis indicates the analyzed genome, and the vertical axis indicates the number of corresponding genes.

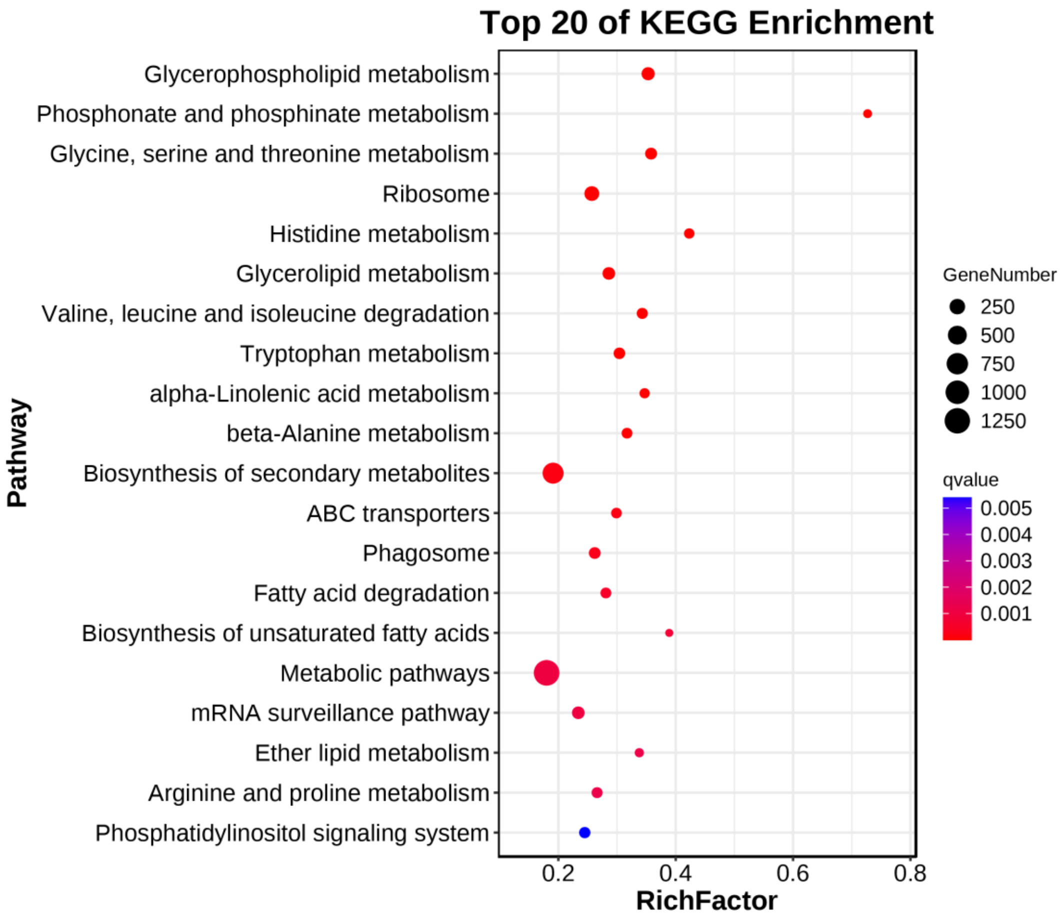

Fig. S7 Significant KEGG pathways enriched in specific genes of *B*. *rapa* W7-2.

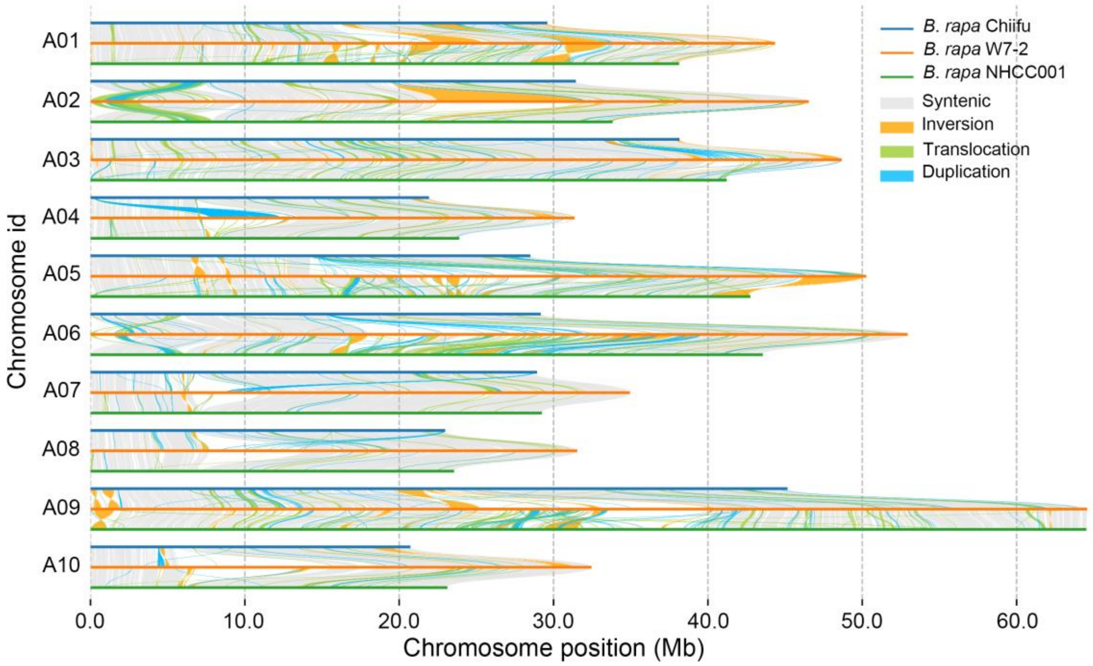

Fig. S8 Synteny analysis based on the comparison of the 10 chromosomes assembled for *B*. *rapa* W7-2 and other *B*. *rapa* sepecies.

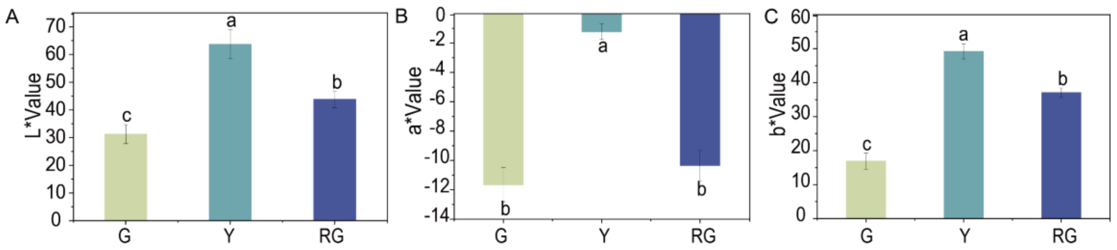

Fig. S9 Changes in the color L*value, a*value, and b*value in G, Y and RG (B).

Data were derived from ten biological replicates at each stage. Error bars represent ± SD. Different letters indicate significant differences ($p < 0.05$). To identify whether low temperature is the key factor causing yellowing of newly emerged inner leaves, we performed a set of temperature treatments from normal temperature to low temperature and then back to normal temperature. At normal temperature, wucai leaves showed the green phenotype (G). When transferred to low temperature for 12 days, the inner leaves exhibited a yellowish color (Y). Then, when the plants were returned to normal temperature, the inner leaves returned to green (RG) (Fig. 3A). As the temperature dropped, the value of L* (lightness), value of a* (redness and greenness) and value of b* (yellowness and blueness) markedly increased. In the Y stage, a* increased from -11.65 to -1.23, b* increased from 16.93 to 49.22, and L* increased from 31.22 to 63.66. As the temperature increased, the values of L*, a* and b* gradually decreased. In the RG stage, the value a* decreased from -1.23 to -10.35, and the value b* decreased from 49.22 to 37.06. There was also a significant difference in the value of L*.

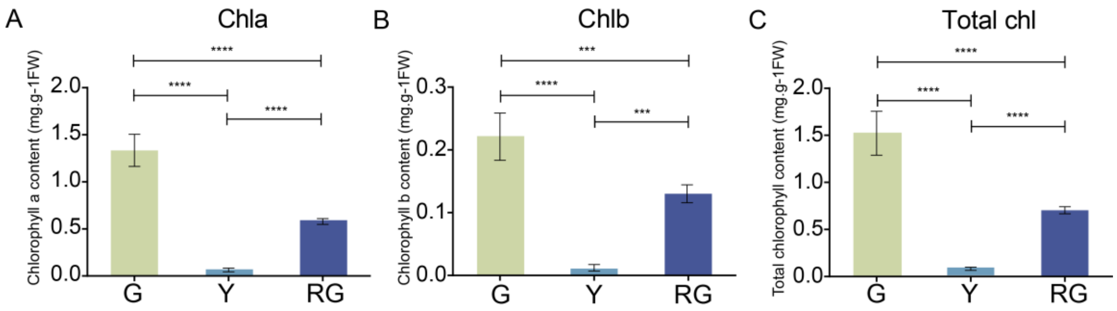

Fig. S10 The Chl content during the three periods G, Y, and RG. The contents of Chl a (A), Chl b (B), and total Chl (C) in inner leaves.

Data were derived from six biological replicates for each stage. Error bars represent ± SD. Different numbers of * above the bars indicate significant differences (***, $p<0.001$, ****, $p<0.0001$). The Chl content in leaves was also evaluated corresponding to three stages. Compared to that in the G stage, the total Chl content in the Y stage decreased significantly, while it obviously increased when plants were returned to normal temperature (RG stage). This indicates that the variation in leaf color in wucai can be mainly attributed to Chl metabolism.

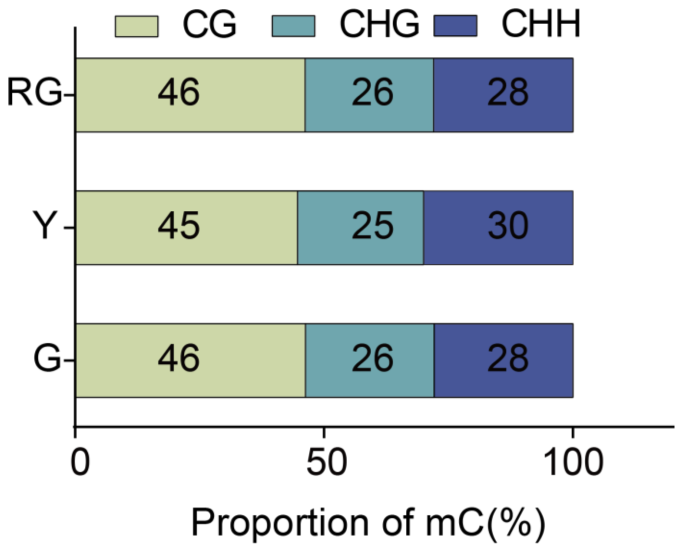

Fig. S11 Proportions of mC in three contexts (CG, CHG, and CHH) in wucai.

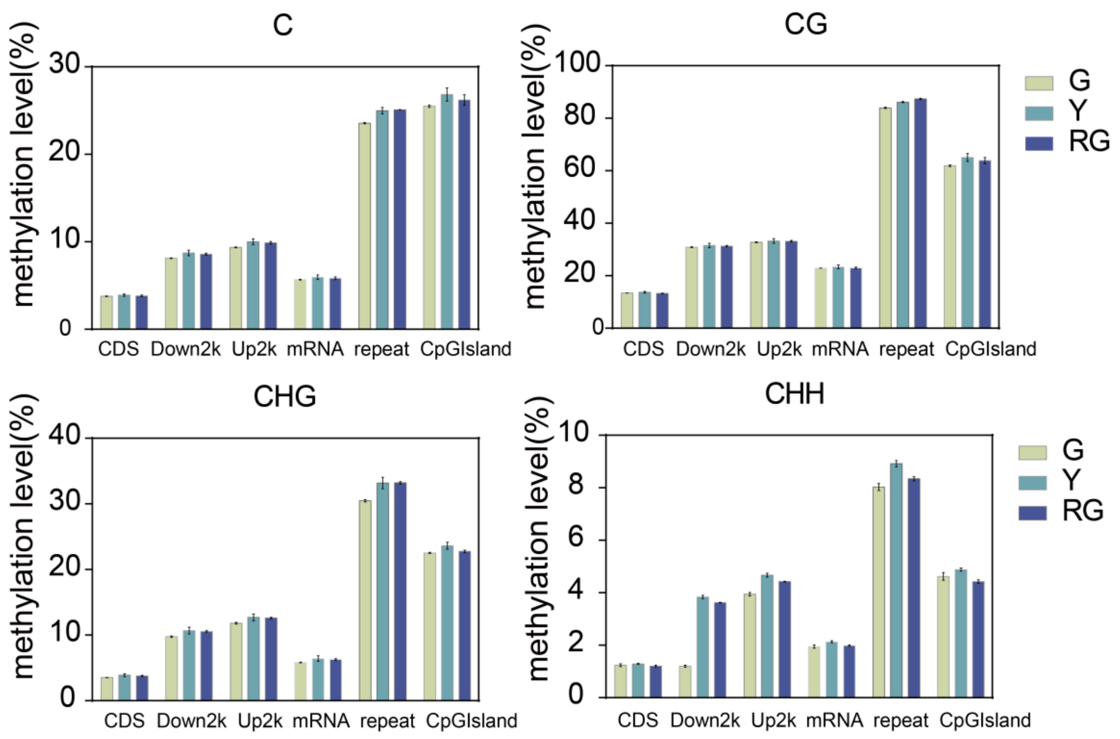

Fig. S12 Average methylation level of mC, mCG, mCHG, and mCHH in some genomic elements.

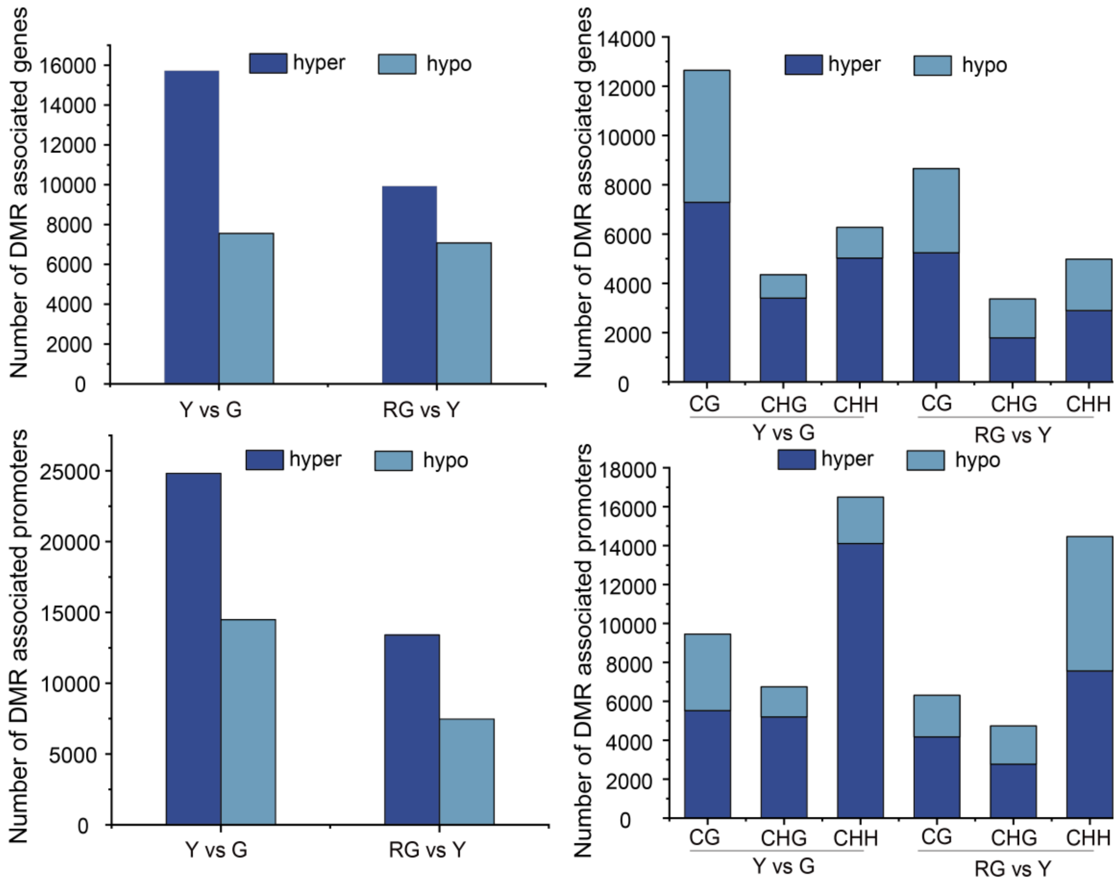

Fig. S13 Numbers of hyper-differentially methylated regions (hyper-DMRs) and hypo-DMRs associated genes and promoters in Y/G and RG/Y.

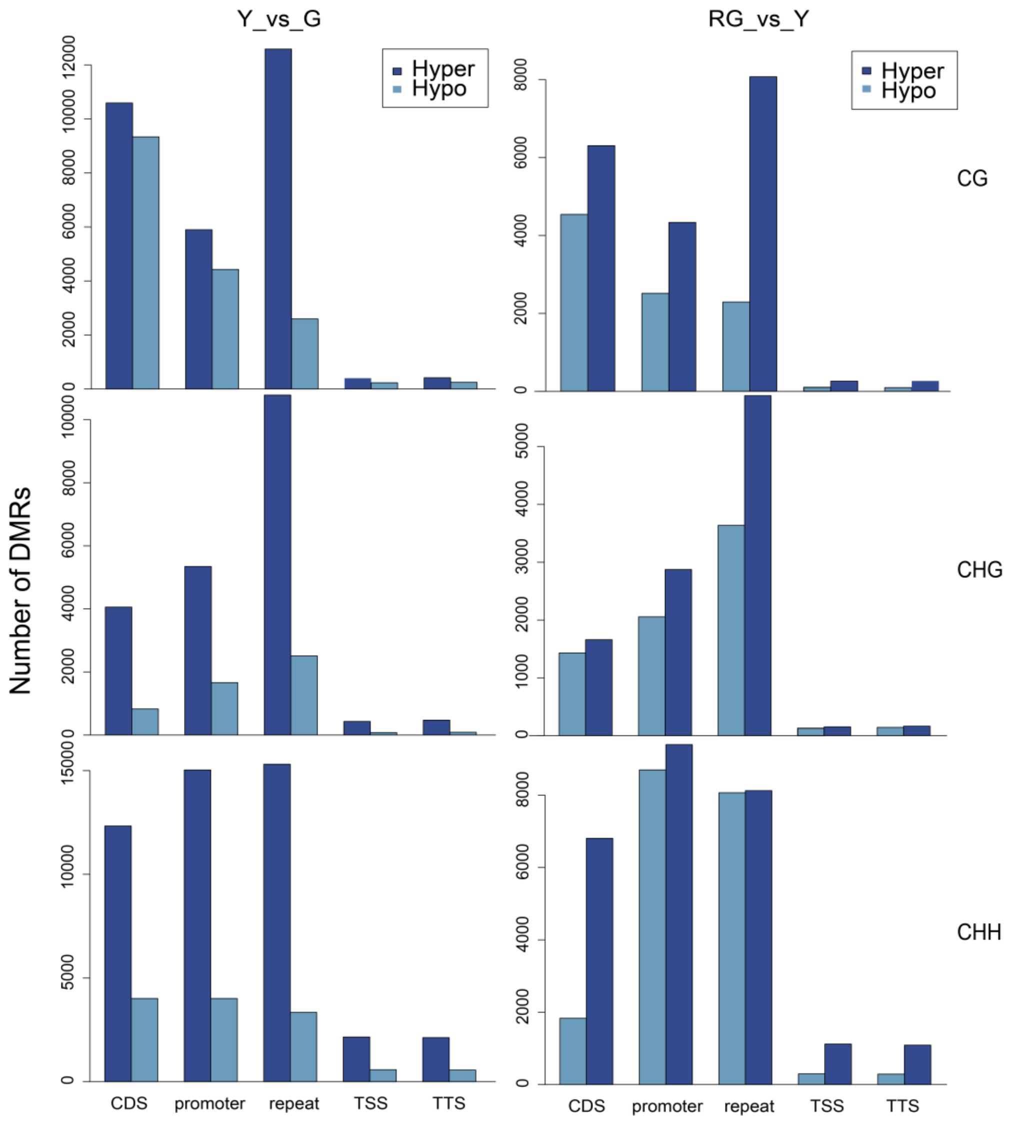

Fig. S14 Distribution of hyper-DMRs and hypo-DMRs in Y/G and RG/Y under mCG, mCHG and mCHH contexts. The abbreviations used are as follows: TSS, transcription start site; TTS, transcription termination site.

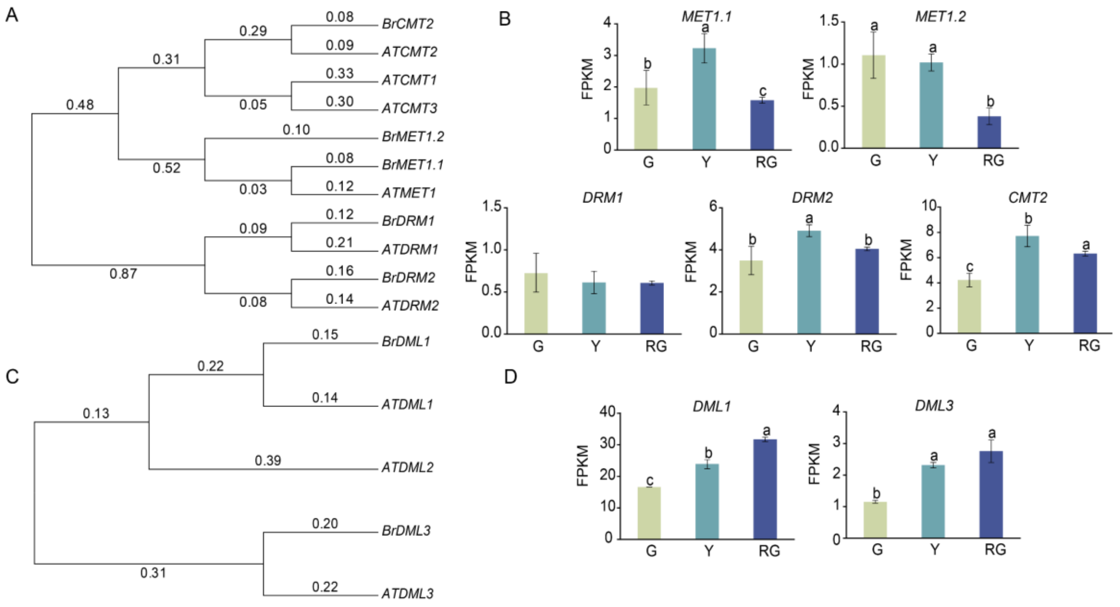

Fig. S15 Phylogenetic analysis and expression of DNA methyltransferase genes and DNA demethylasegenes (DML) in G, Y, and RG. (A) Phylogenetic analysis of DNA methyltransferase in wucai and Arabidopsis. (B) Transcript levels of DNA methyltransferase genes in three stages. (C) Phylogenetic analysis of DNA demethylase in wucai and Arabidopsis. (D) Transcript levels of DNA demethylase genes in three periods. Error bars represent ± SD. Different letters indicate significant differences ($p<0.05$).

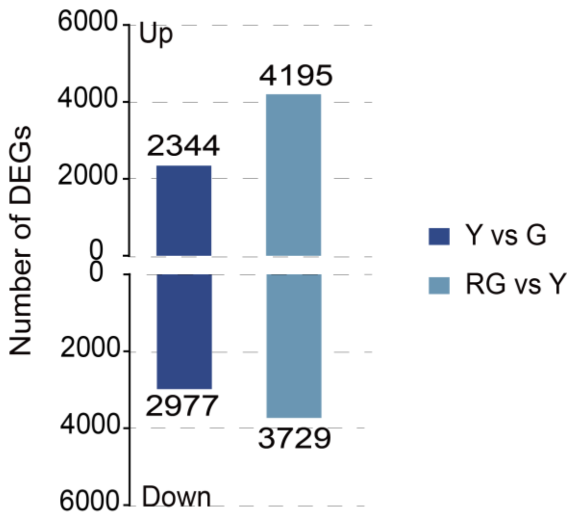

Fig. S16 The number of DEGs was obtained by comparing Y/G and RG/Y.

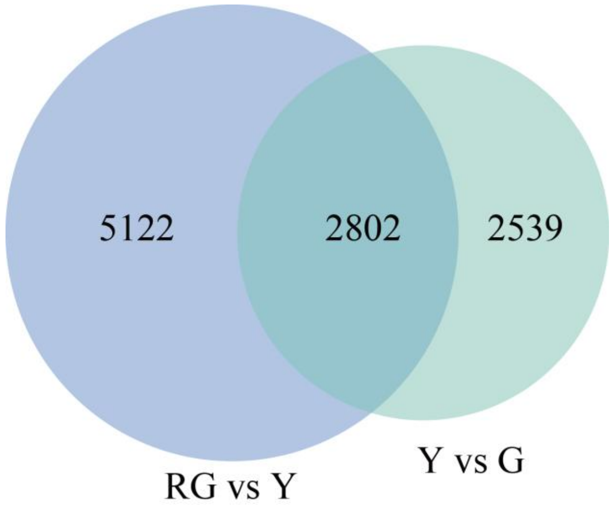

Fig. S17 Venn diagram of the resulting DEGs for Y/G and RG/Y comparisons.

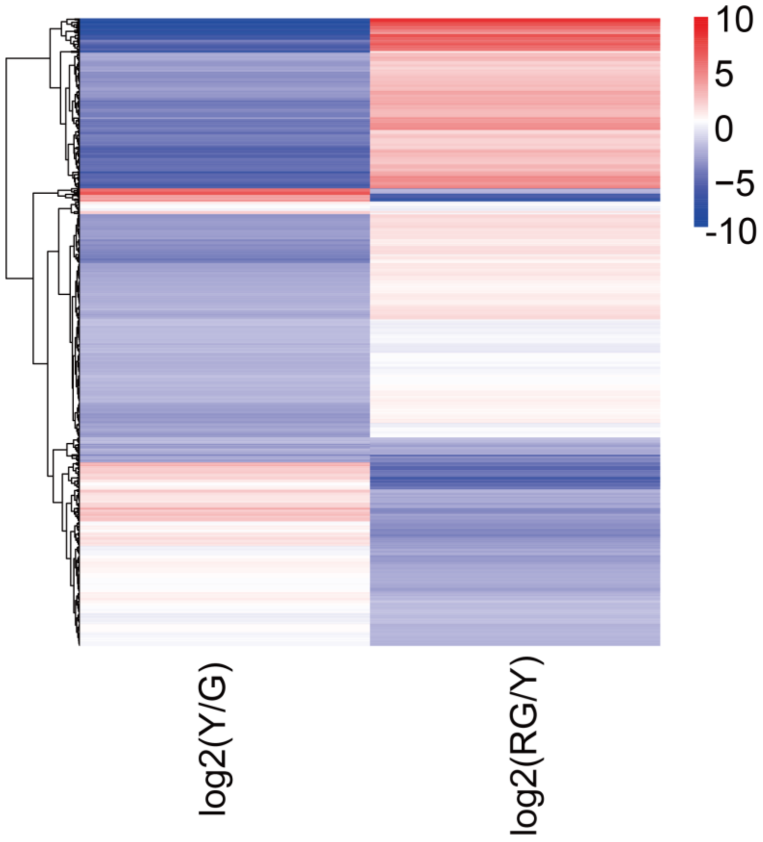

Fig. S18 Hierarchical clustering of DEGs co-expressed between Y/G and RG/Y. Rows in the heat map represent genes and columns represent samples. The color of the heat map indicates the gene expression level [logz fragment per millionth kilobase (FPKM)] of the different samples. Color gradients, from blue to white to red, represent low, medium, and high values of gene expression.

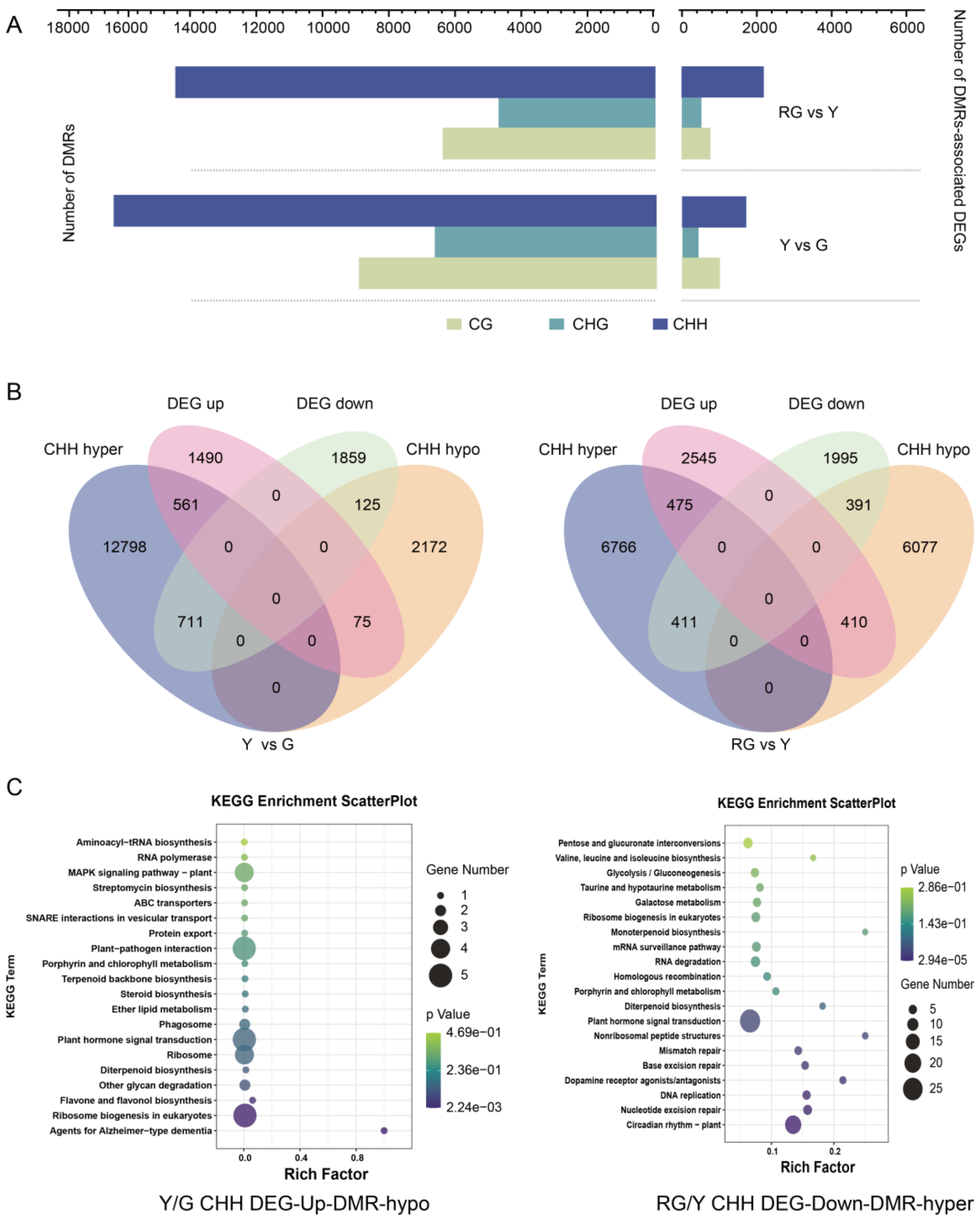

Fig. S19 (A)The number of DMRs and DMRs-associated DEGs in Y/G and RG/Y. (B) The Venn diagram shows the number of DMRs and DMRs-associated DEGs in CHH. **C** KEGG annotates the DMRs related DEGs pathway between Y/G and RG/Y.

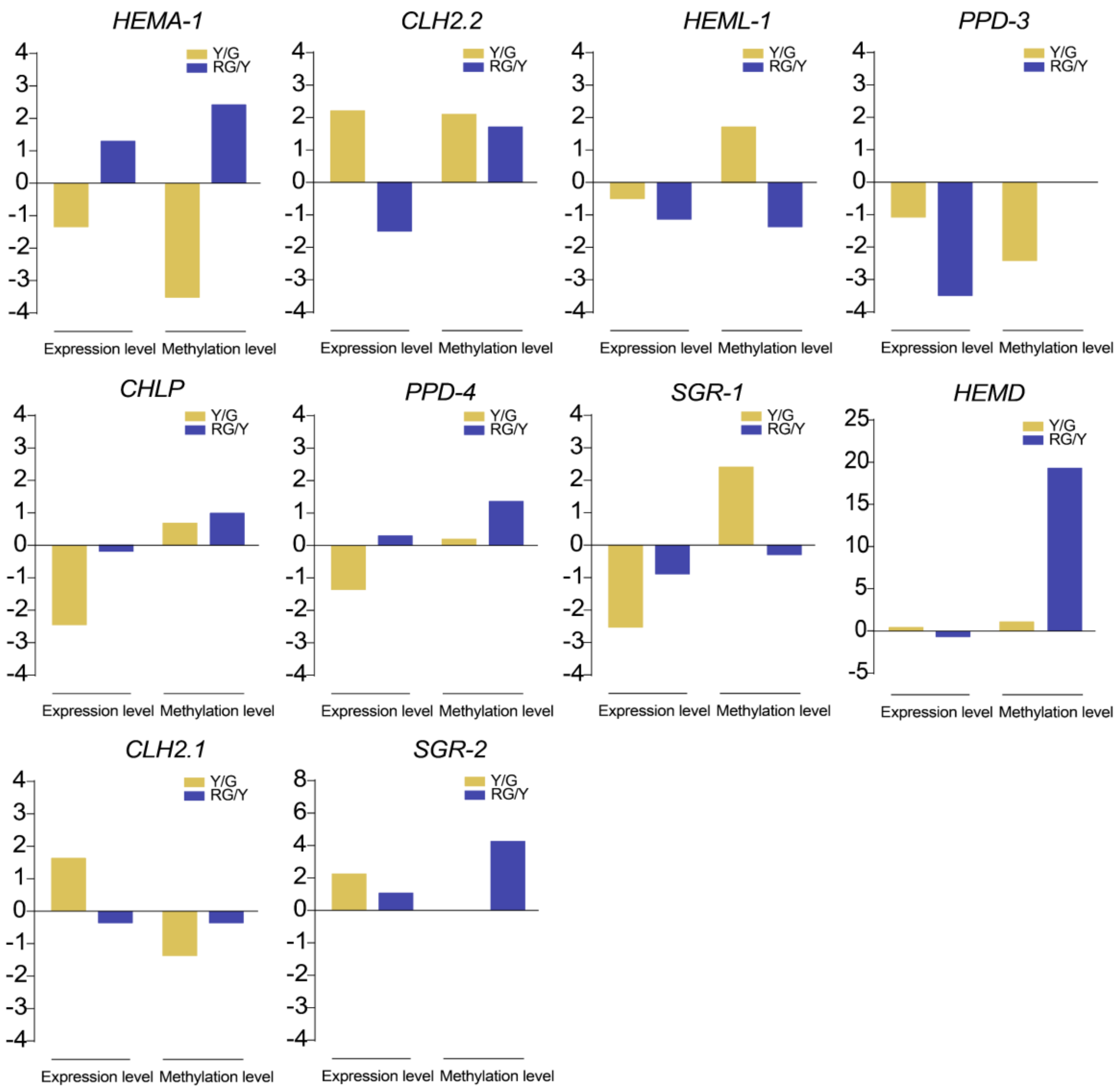

Fig. S20 Correlation analysis between DNA methylation levels and expression levels in chlorophyll metabolism during inner-leaf yellowing in wucai.

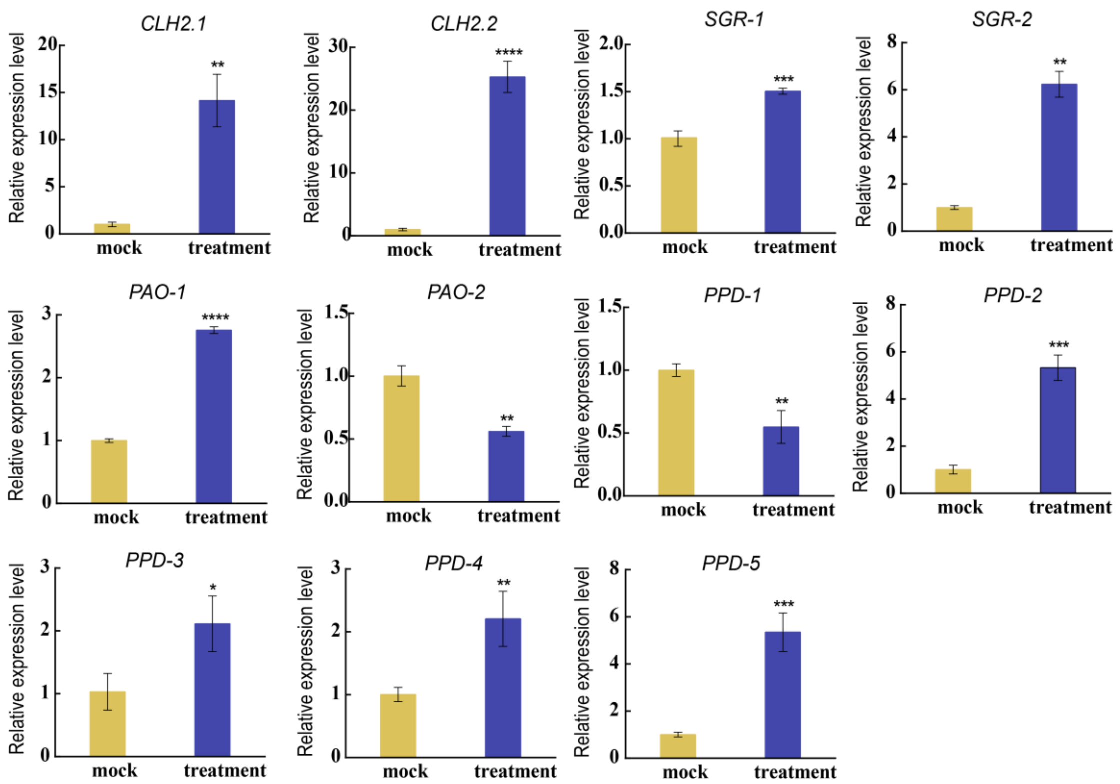

Fig. S21 The expression of chlorophyll degradation-related genes in mock and 100-Aza treated wucai inner-leaf. Data were derived from six biological replicates of each period. Error bars represent ± SD. Different letters indicate significant differences (p<0.05). Different numbers of * above the bars indicate significant differences (*, p<0.05, **, p<0.01, ***, p<0.001, ****, p<0.0001).

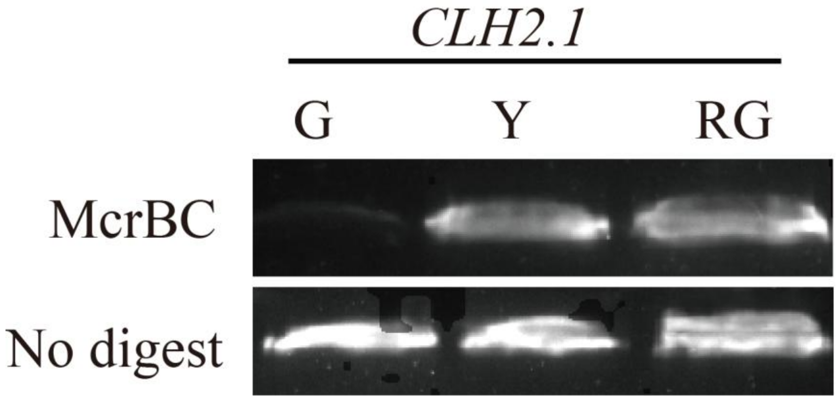

Fig. S22 Analysis of promoter methylation levels of *BrCLH2.1* in three stages.
