# Supplementary material for: The wucai genome and DNA methylation regulation on the inner leaves’ yellowing response to low temperature
Source: Hortic Res. 2025 Sep 2;12(12):uhaf231. doi: 10.1093/hr/uhaf231 (PMC12680381; doi:10.1093/hr/uhaf231)
Supplement: Web_Material_uhaf231 [file web_material_uhaf231.zip › Revised supplementary Tables.pdf]

Supplemental Tables

Table. S1 Survey filter data statistics

Table. S2 *K*-mer statistic results of *B. rapa* W7-2 genome

Table. S3 HiFi sequencing data statistics

Table. S4 Statistics of Hi-C assembly results

Table. S5 Length of each super scaffold of assembly result

Table. S6 Quality assessment of the assembled genome using BUSCO

Table. S7 Comparisons of assembly statistics between the B. rapa W7-2 genome and other published B. rapa genome versions

Table. S8 Summary of the gene functional annotations in *B. rapa* W7-2 genome

Table. S9 Non-coding RNAs predicted from the *B. rapa* W7-2 genome

Table. S10 Summary of isoform clusters

Table. S11 Summary of gene-isoforms classification in each sample

Table. S12 Summary of transcripts classification in each sample

Table. S13 Statistics of repeat contents in the assembled genome.

Table. S14 Summary of transposable elements in B. rapa W7-2 genome

Table. S15 Statistics of gene annotation completeness in the assembled genome using BUSCO

Table. S16 The statistics of gene family in the B. rapa W7-2 and other 18 plant genomes.

Table. S17 The statistics of structural variation annotations between the genomes of *B. rapa* Chiifu and *B. rapa* W7-2.

Table. S18 The statistics of sequence variation annotations between the genomes of *B. rapa* Chiifu and *B. rapa* W7-2.

Table. S19 The statistics of structural variation annotations between the genomes of *B. rapa* W7-2 and *B. rapa* NHCC001.

Table. S20 The statistics of sequence variation annotations between the genomes of *B. rapa* W7-2 and *B. rapa* NHCC001.

Table. S21 Data Summary and QC

Table. S22 Alignment statistics with reference genome

Table. S23 Numbers of hyper-differentially methylated regions (hyper-DMRs) and hypo-

DMRs associated genes and promoters in Y/G and RG/Y.

Table. S24 Quality of RNA-seq data.

Table. S25 The overlapping genes of DEGs and CHH-DMR genes in Y/G and RG/Y.

Table. S26 Primer sequences for RT-qPCR.

Table. S1 Survey filter data statistics

Lib ID	Insert Size(bp)	Read Length(bp)	Raw Data (Gb)	Clean Data (Gb)	GC (%)
W7-2	300-400	150	64.01	62.96	37.96

Table. S2 *K*-mer statistic results of *B*. *rapa* W7-2 genome

kmer	nkmer	usedbase	heter	genome size	repeat	repeat rate	err rate	seqdepth
17	53782583562	62662205620	0.005	466912887	301646135	0.646	0.0048	134.21
19	53245834673	62632658472	0.0047	468687886	269763118	0.5756	0.0052	133.63
21	52668283338	62630147572	0.0044	470693033	258987291	0.5502	0.0053	133.06
23	52060109460	62633756519	0.0042	472735588	253968590	0.5372	0.0052	132.49
25	51427626472	62642091347	0.0039	474911673	250876487	0.5283	0.0051	131.9
27	50767132075	62646581123	0.0037	477247343	248736014	0.5212	0.005	131.27
29	50085147524	62650933014	0.0036	479444553	246966392	0.5151	0.0049	130.67
31	49382705290	62655535491	0.0034	481107218	245016620	0.5093	0.0049	130.23

Table. S3 HiFi sequencing data statistics

	Raw data base (G)	Valid read	Valid read base (G)	Clean data N50
1	213.14	117480	1.54	13533
2	295.06	1029115	15.92	16505

Table. S4 Statistics of Hi-C assembly results

Assembly	Scaffold		Contig	
	Length (bp)	Number	Length (bp)	Number
Max length	64567558		19941482	
N10	64567558	1	17189345	3
N20	52943294	2	9206775	7
N30	50256853	3	6791951	13
N40	48667824	4	6009131	20
N50	46534226	5	4450120	30
N60	44347353	6	3324840	42
N70	34949166	7	2055667	61
N80	31530805	9	1471373	88
N90	31358815	10	761289	133
Total length	480669177		480568177	
Total number		303		505
GC rate	37.40%		37.40%	

Table. S5 Length of each super scaffold of assembly result

Scaffold	Length
HiC scaffold 1	44347353
HiC scaffold 2	46534226
HiC scaffold 3	48667824
HiC scaffold 4	31358815
HiC scaffold 5	50256853
HiC scaffold 6	52943294
HiC scaffold 7	34949166
HiC scaffold 8	31530805
HiC scaffold 9	64567558
HiC scaffold 10	32459865

Table. S6 Quality assessment of the assembled genome using BUSCO

Type	Number
Complete BUSCOs ©	1603
Complete and single-copy BUSCOs (S)	1266
Complete and duplicated BUSCOs (D)	337
Fragmented BUSCOs (F)	4
Missing BUSCOs (M)	7
Total BUSCO groups searched	1614

Table S7 Comparisons of assembly statistics between the B. *rapa* W7-2 genome and other published B. *rapa* genome versions

	W7-2<br>This study	PC-fu<br>(Xu et *al*., 2022)	NHCC001<br>(Li et al., 2020)	Purple pakchoi<br>(Li et al., 2021)	Chiifu (v 4.0)<br>(Zhang et al., 2023)
Assembly genome size (Mb)	480.57	411.4	405.33	370.42	424.59
GC content (%)	37.4	37.68	37.13	37.12	37.59
Number of contig	505	2288	602	1985	12
Contig N50 (Mb)	4.45	4.7	2.83	2.82	38.26
Scaffold N50	46.53 Mb	39389 bp	38.13Mb		
TE length (Mb)	247.50 Mb	260.3 Mb	187.1 Mb	147.09 Mb	228.35 Mb
TE rate (% of genome)	51.49%	63.30%	46.15%	39.66%	53.78%
BUSCO- assembly	1614	1440	1614	1440	
Complete BUSCO	99.30%	99.20%	99.07%	98.10%	99.40%
Protein-coding genes	42,548	52,511	48,158	45,363	47,531
Average gene length (bp)		2220	2119	1125	
BUSCO—annotation	92.69%	98.20%		96.60%	

Table. S8 Summary of the gene functional annotations in *B. rapa* W7-2 genome

Values	Number	Percentage
Total	42,634	100%
Nr-Annotated	42,541	99.78%
Swissprot-Annotated	35,965	84.36%
KEGG-Annotated	32,948	77.28%
KOG-Annotated	33,985	79.71%
TrEMBL-Annotated	42,479	99.64%
Interpro-Annotated	40,305	94.54%
GO-Annotated	26,553	62.28%
Overall	42,548	99.80%

Table. S9 Non-coding RNAs predicted from the *B. rapa* W7-2 genome

Type		Copy (w)	Average length (bp)	Total length (bp)	Ratio in the genome (%)
miRNA		219	127.7579909	27979	0.005821
tRNA		2320	74.86465517	173686	0.036134
rRNA	rRNA	20055	314.5729743	6308761	1.312495
	18S	2570	1375.71751	3535594	0.735557
	28S	7836	133.976391	1049839	0.218412
	5.8S	2013	388.7968207	782648	0.162825
	5S	7636	123.1901519	940680	0.195702
snRNA	snRNA	1526	110.7601573	169020	0.035163
	CD-box	1219	104.2026251	127023	0.026426
	HACA-box	119	127.8319328	15212	0.003165
	splicing	188	142.4734043	26785	0.005572

Table. S10 Summary of isoform clusters

Sample	Total isoforms	Total base(bp)	Mean Quality	Mean isoforms length(bp)	Mean Full length coverage
W7-2	469,227	504,296,787	0.98	1,075	3.00

Table. S11 Summary of gene-isoforms classification in each sample

Sample	Total RefGene	Detected RefGene	Ratio of Total RefGene	Mean isoforms per Gene
W7-2	41,234	27,894	67.65%	12.67

Table. S12 Summary of transcripts classification in each sample

Sample	Unique isoforms	Known transcripts	Alternative splicing events	Novel transcripts	Other type
W7-2	78,918	27,894	26,693	25,385	16,499

Table. S13 Repetitive elements predicted from the *B. rapa* W7-2 assembly

Type	Repeat Size (bp)	Ratio in the genome (%)
Trf	102083760	21.237842
Repeatmasker	61250822	12.742823
Proteinmask	45707265	9.50909
De novo	247502395	51.491214
Total	264759976	55.081538

Table. S14 Summary of transposable elements in *B. rapa* W7-2 genome

Type	Length (bp)	Ratio in the genome (%)
DNA	35373356	7.35919
LINE	17962906	3.737062
SINE	42940	0.008933
LTR	134435616	27.968429
Other	0	0
Satellite	48849360	10.162782
Simple_repeat	641020	0.13336
Unknown	21316776	4.434812
Total	247502395	51.491214

Table. S15 Statistics of gene annotation completeness in the assembled genome using BUSCO

Type	Number
Complete BUSCOs ©	1496
Complete and single-copy BUSCOs (S)	1161
Complete and duplicated BUSCOs (D)	335
Fragmented BUSCOs (F)	84
Missing BUSCOs (M)	34
Total BUSCO groups searched	1614

Table. S16 The statistics of gene family in the *B. rapa* W7-2 and other 18 plant genomes.

Species	Total	Single-copy genes	Multiple-copy genes	Unique genes	Other genes	unclusternum
A. thaliana	27569	4649	8225	389	13042	1264
B. rapa W7-2	42634	2344	18692	195	20611	792
B. rapa NHCC001	48158	2505	17273	411	27367	602
B. rapa PC-fu	52511	2517	17022	228	32054	690
B. juncea AA	47892	2566	16680	59	28105	482
B. juncea BB	46777	2520	16927	784	24915	1631
B. napus AA	44211	2588	16252	455	23025	1891
B. napus CC	52349	2406	17504	783	29537	2119
B. oleracea	59438	2401	17850	1929	35560	1698
B. rapa Chiifu	45140	2655	16277	205	24973	1030
C. rubella	27681	4628	8371	542	13437	703
C. sativus	27211	5342	6365	2130	8689	4685
I. triloba	31351	3824	11795	2759	10694	2279
L. sativa	38909	3908	11880	7351	12245	3525
M. sativa	49120	4543	10091	13179	17565	3742
O. sativa	42173	4571	8957	12975	7684	7986
R. sativus	40100	2679	15947	924	18727	1823
S. lycopersicum	35343	4242	10348	4157	12149	4447
V. vinifera	31315	4868	8331	2649	11799	3668

Table. S17 The statistics of structural variation annotations between the genomes of *B. rapa* Chiifu and *B. rapa* W7-2.

Structural variations	Counts	Length reference (bp)	Length query (bp)
Syntenic regions	7709	192563514	187317163
Inversions	94	13334533	27198614
Translocations	3959	34994204	35676588
Duplications (reference)	1136	7615974	-
Duplications (query)	17231	-	68858674
Not aligned (reference)	9655	71750243	-
Not aligned (query)	24783	-	121906130

Table. S18 The statistics of sequence variation annotations between the genomes of *B. rapa* Chiifu and *B. rapa* W7-2.

Sequence variations	Counts	Length reference (bp)	Length query (bp)
SNPs	1173726	1173726	1173726
Insertions	265138	-	2888906
Deletions	123104	3433062	-
Copygains	304	-	2650367
Copylosses	457	2053679	-
Highly diverged	17199	47629865	56866920
Tandem repeats	34	240993	292043

Table. S19 The statistics of structural variation annotations between the genomes of *B. rapa* W7-2 and *B. rapa* NHCC001.

Structural variations	Counts	Length reference (bp)	Length query (bp)
Syntenic regions	6462	217837884	208930467
Inversions	134	26970157	23579915
Translocations	4008	40947578	41148203
Duplications (reference)	692	8876502	-
Duplications (query)	5675	-	25926508
Not aligned (reference)	9193	155822214	-
Not aligned (query)	13399	-	71359421

Table. S20 The statistics of sequence variation annotations between the genomes of *B. rapa* W7-2 and *B. rapa* NHCC001.

Sequence variations	Counts	Length reference (bp)	Length query (bp)
SNPs	1082335	1082335	1082335
Insertions	292234	-	3063839
Deletions	140027	3951464	-
Copygains	265	-	1926521
Copylosses	406	3294266	-
Highly diverged	15200	55928155	45975873
Tandem repeats	63	512076	533883

Table. S21 Data Summary and QC

Sample ID	Clean Reads Q20 Rate (%)	Clean Data Size (bp)	Mapping Rate (%)	Bisulfite Conversion Rate (%)	Duplication Rate (%)	Average Depth (X)	Coverage (%)
G1	96.67;95.57 (Pass)	25062971700	91.27 (Pass)	99.38 (Pass)	2.67 (Pass)	26.53	87.594
G2	96.51;95.15 (Pass)	20994950700	91.56 (Pass)	99.44 (Pass)	2.25 (Pass)	22.45	87.297
G3	96.68;93.54 (Pass)	23678172600	90.70 (Pass)	99.48 (Pass)	2.54 (Pass)	25.04	87.368
Y1	96.37;93.32 (Pass)	16972063800	90.52 (Pass)	99.51 (Pass)	2.41 (Pass)	17.42	86.69
Y2	96.34;93.01 (Pass)	18587526600	90.65 (Pass)	99.50 (Pass)	2.29 (Pass)	18.78	86.175
Y3	96.18;93.35 (Pass)	30423502800	90.56 (Pass)	99.45 (Pass)	3.48 (Pass)	31.08	88.811
RG1	97.30;97.12 (Pass)	18978333900	93.15 (Pass)	99.47 (Pass)	2.19 (Pass)	20.5	86.858
RG2	97.43;98.47 (Pass)	16602303300	92.68 (Pass)	99.51 (Pass)	2.06 (Pass)	18.15	86.403
RG3	97.19;98.24 (Pass)	16542113100	92.68 (Pass)	99.24 (Pass)	1.88 (Pass)	18.3	86.557

Table. S22 Alignment statistics with reference genome

Sample ID	Clean Reads	Mapped Reads	Mapping Rate (%)	Uniquely Mapped Reads	Uniquely Mapping Rate (%)	Bisulfite Conversion Rate (%)
G1	167086478	152500157	91.27	84850233	50.78	99.38
G2	139966338	128156595	91.56	71706327	51.23	99.44
G3	157854484	143181242	90.7	80163316	50.78	99.48
Y1	113147092	102422727	90.52	55767530	49.29	99.51
Y2	123916844	112329944	90.65	60073781	48.48	99.5
Y3	202823352	183676502	90.56	100259740	49.43	99.45
RG1	126522226	117851415	93.15	65554749	51.81	99.47
RG2	110682022	102576421	92.68	57983287	52.39	99.51
RG3	110280754	102213712	92.68	58389949	52.95	99.24

Table. S23 Numbers of hyper-differentially methylated regions (hyper-DMRs) and hypo-DMRs associated genes and promoters in Y/G and RG/Y.

	Y/G		RG/Y	
gene DMRS	hyper	hypo	hyper	hypo
CG	7322	5327	5253	3503
CHG	3378	970	1797	1588
CHH	4925	1196	2974	2078
Total DMRS	15625	7493	10024	7169
Total	23118		17193	

	Y/G		RG/Y	
pro DMRS	hyper	hypo	hyper	hypo
CG	5517	3949	4197	2169
CHG	5144	1575	2767	1959
CHH	14070	2372	7652	6878
Total DMRS	24731	7896	14616	11006
Total	32627		25622	

Table. S24 Quality of RNA-seq data

Sample_ name	Total_raw _read	Total_clea n_read	Total_clea n_base	Clean_rea d_q20	Clean_rea d_q30	Clean_read _ratio
G1	43.3	43.3	6.49	97.86	94.04	100
G2	42.37	42.37	6.36	97.97	94.38	100
G3	42.19	42.19	6.33	98.07	94.6	100
Y1	42	42	6.3	97.81	93.87	100
Y2	42.45	42.45	6.37	97.86	94.09	100
Y3	43.54	43.54	6.53	97.94	94.25	100
RG1	43.41	43.41	6.51	97.86	94.07	100
RG2	43.43	43.43	6.51	97.84	93.97	100
RG3	43.28	43.28	6.49	97.35	91.95	100

Table. S25 The overlapping genes of DEGs and CHH-DMR genes in Y/G and RG/Y

Samples	DEG-up genes number		DEG-down genes number		Total genes number
	DMR-hypo genes number	DMR-hyper genes number	DMR-hypo genes number	DMR-hyper genes number	
Y/G	75	561	125	711	1472
RG/Y	410	475	391	411	1687

Table. S26 Primer sequences for RT-qPCR.

Gene name	Primer name	Primer sequence (5’->3’)
BrActin	primer F	TGGGTTTGCTGGTGACGAT
	primer R	TGTCTAGGACGACCAACAATACT
BrCLH2.1	primer F	GCTTGATGATGATACTGAAGGGA
	primer R	ATAAGCCATCAAAAACGCCAC
BrCLH2.2	primer F	TTGCTGAGTGGAAGAAGGTGA
	primer R	AGATCACAACTTTTAGCCCCA
BrSGR-1	primer F	CGTTGCTAAGACTATGAGACC
	primer R	TGAGAAACAGTGCGTGGATGA
BrSGR-2	primer F	GCTTCCGTCTCTATCATCCCT
	primer R	TGCCATGTGCCAGTATCCTTC
BrPPD-1	primer F	GCTTCCGTCTCTATCATCCCT
	primer R	TGCCATGTGCCAGTATCCTTC
BrPPD-2	primer F	AGAAGCAAGAGAGTAAGTCGT
	primer R	AACGGACCCATCGAATCT
BrPPD-3	primer F	CATCAACCTCACCGACGCTAA
	primer R	ATCGGAGAGGAGAGAGAAGAG
BrPPD-4	primer F	TTAGGGCTTTGAACGGTGCTG
	primer R	AGTCTTGATGTAAACGCGAGG
BrPPD-5	primer F	CATCCATCAGACTCAGCTTT
	primer R	GCTCTCTGATGAAAAATCTGGT

BrPAO-1	primer F	GGAGTTGTGTGGGTTTGGA
	primer R	ACCCGAGATGTCATAGAAACC
BrPAO-2	primer F	TCCCAACCAAATCAGACGCA
	primer R	ATACTTCCCTCTCCAATCGAC
BrCMT2	primer F	AATGTCCTATGAAAGTCTCCC
	primer R	CAGTAGCATCGTCCTTTTTGT
BrDRM2	primer F	TAACAGAGTGAGCAGGAGTGG
	primer R	CGTCTTTGTTGTTTCTCGCACC
